# Biohybrid Computing with Proteinoids and Algae

**DOI:** 10.1002/advs.202506155

**Published:** 2025-07-13

**Authors:** Panagiotis Mougkogiannis, Andrew Adamatzky

**Affiliations:** ^1^ Unconventional Computing Laboratory University of the West of England Bristol BS16 1QY UK

**Keywords:** algae, biohybrid systems, protein‐like polymers, proteinoids, self‐assembly, thermal synthesis, unconventional computing

## Abstract

Proteinoids, or thermal proteinoids, are produced by heating amino‐acids. When placed in water, proteinoids swell into microspheres which produce neuron‐like spikes of electrical potential. This study combines proteinoid microspheres with *Emiliania huxleyi* algae to create advanced bioelectronic signal processing systems with neuromorphic characteristics. The morphologies of L‐Glu:L‐Phe proteinoid microspheres and their interactions with algae are studied using scanning electron microscopy, revealing complex structures with budding forms and traits of self‐assembly. Electrical measurements demonstrate that both pure algae and algae‐proteinoid mixtures generate spontaneous oscillations with unique amplitude and frequency patterns across different recording channels. The algae‐proteinoid mixture exhibits a broad range of oscillatory dynamics with amplitudes varying from 25.49 to 191.42 mV and periods ranging from 251.92 to 5471.01 s. These oscillatory behaviors are utilized to perform Boolean logic operations, through post‐processing of biological signals rather than autonomous computing, with pure algae systems showing superior performance for direct gates (AND, OR) while the mixture excels at inverse gates (NAND, NOR). Temperature and pH emerge as critical factors controlling oscillatory dynamics. The findings indicate that algae‐proteinoid electrochemical systems represent a step toward biohybrid computing and offer a sustainable and biocompatible alternative for unconventional computing, providing enhanced signal stability, environmental resilience, and more effective information processing compared to traditional electronic systems while acknowledging current limitations in autonomous learning capabilities.

## Graphical abstract

We developed a novel biohybrid computing system by combining proteinoid microspheres with algae. The biological mixture creates spontaneous electrical oscillations. These oscillations can perform simple logic operations, such as AND, OR, NAND, and NOR gates. Environmental factors like temperature and pH affect how the system works. They provide a sustainable option for unconventional computing applications, unlike traditional electronics.

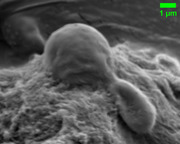

## 1 Introduction

Bio‐inspired materials are gaining significant interest for advanced electronics applications.^[^ ^1^, ^2^, ^3^ ^]^ This growing interest stems from researchers' pursuit of sustainable and biocompatible alternatives to traditional semiconductor materials, driven by both environmental concerns and the increasing demand for technologies that seamlessly integrate with biological systems.^[^ ^4^, ^5^, ^6^, ^7^, ^8^, ^9^, ^10^, ^11^, ^12^, ^13^ ^]^

Proteinoid‐based composites stand out among these materials due to their remarkable ability to self‐assemble, structural diversity, and capacity to mimic biological functions.^[^ ^14^, ^15^, ^16^, ^17^ ^]^ These unique materials possess extraordinary properties that position them as potential revolutionaries in next‐generation electronics, serving as crucial bridges between synthetic and natural systems.^[^ ^18^, ^19^, ^20^ ^]^

The integration of algal components with proteinoid matrices represents a groundbreaking approach to hybrid material fabrication, creating systems capable of exhibiting neuron‐like spiking behavior.^[^ ^21^, ^22^, ^23^ ^]^ This synergistic combination not only enhances material performance but also establishes new pathways for sustainable bioelectronics development.^[^ ^24^, ^25^, ^26^ ^]^

Proteinoids exhibit extraordinary structural versatility through their ability to form dynamic oligopeptide structures via thermal amino acid polymerization and subsequently self‐assemble into complex, larger aggregates.^[^ ^27^, ^28^, ^29^, ^30^ ^]^ This remarkable capacity to generate sophisticated, biologically inspired architectures positions them as fundamental components for next‐generation bioelectronic devices and future technological innovations.

The versatility of proteinoid manufacturing techniques enables diverse fabrication approaches, each tailored to specific applications ranging from fundamental scientific research to advanced biomedical engineering.^[^ ^31^, ^32^, ^33^ ^]^ This manufacturing flexibility provides an exceptional platform for systematic material design and optimization, facilitating rapid adoption across multiple disciplines.^[^ ^34^, ^35^, ^36^, ^37^, ^38^ ^]^

Within the context of neuromorphic engineering and bioelectronics, our approach involves the strategic combination of biological components (algae) with synthetic materials (proteinoids) to create functional interfaces capable of electrical signal processing.^[^ ^39^, ^40^, ^41^ ^]^ While this represents a significant advancement toward biohybrid computing systems, it differs from fully autonomous neuromorphic systems that demonstrate independent learning and adaptation capabilities.^[^ ^42^, ^43^, ^44^, ^45^ ^]^This system exploits intrinsic biological oscillations to perform basic computational functions through signal processing, representing an important technological milestone as the field progresses toward more advanced bio‐inspired computing paradigms.

Neuron‐like spiking in proteinoid microspheres is similar to action potentials in neurons.^[^ ^17^, ^46^, ^47^, ^48^, ^49^ ^]^ This similarity highlights the potential of these materials as key parts in bio‐inspired electronic devices. Proteinoids' ability to mimic neural behavior positions them as transformative elements in neuromorphic engineering, enabling the development of brain‐like devices with enhanced efficiency and performance. The spiking activity observed in proteinoids represents an active, energy‐dependent process involving ionic transport across proteinoid structures that closely parallels natural neural signaling mechanisms.^[^ ^50^ ^]^ This dynamic process demonstrates the complex interplay between physical and chemical forces within proteinoids, providing fundamental insights into the principles of biological computation.^[^ ^51^, ^52^ ^]^ The incorporation of algal components into proteinoid matrices introduces additional functional dimensions to these materials, potentially enhancing their electronic and optical properties while simultaneously improving biocompatibility and sustainability. This synergistic algae‐proteinoid combination not only elevates material performance but also advances global initiatives toward environmentally sustainable technologies.^[^ ^53^, ^54^, ^55^ ^]^

The thermal proteins can create microspheres that show various biologically inspired responses.^[^ ^56^, ^57^ ^]^ We can change the electronic properties of these materials by interacting with chemicals like chloroform.^[^ ^58^ ^]^ This could lead to new uses in biological computing. This tunability shows how these materials can change the game for adaptive systems in new computing methods. We can control how the organic parts interact with the proteinoid matrix. By fine‐tuning the fabrication process, we can customize the material's properties precisely. This precision engineering allows for new ways to design materials with specific functions. It is revolutionizing bioelectronic applications.

This work centers on proteinoid‐algae interfaces that generate oscillating electrical signals suitable for fundamental computational operations, including Boolean logic functions that serve as building blocks for traditional computing architectures.

Studying proteinoid oscillations gives us important insights into how molecules behave and interact. This knowledge is key for understanding how life began and for exploring the chance of alien life.^[^ ^59^, ^60^ ^]^ These oscillations offer a unique window into the origins of life while revealing the dynamic behavior of proteinoids, demonstrating their high sensitivity and adaptability to environmental changes—characteristics that carry profound implications for astrobiology and synthetic biology and represent essential features for real‐world applications.^[^ ^61^, ^62^, ^63^, ^64^, ^65^ ^]^

Oscillations in prebiotic environments are now seen as important for chemical evolution and the origin of life. This idea is backed by several key studies. Damer et al. introduce the Hot Spring Hypothesis.^[^ ^61^ ^]^ They show that wet‐dry cycles in places like hot springs helped create and concentrate organic molecules. These cycles involve changes in temperature, pH, and hydration. Their experiments show how changing conditions may have led to protocell formation. This gives us a strong basis to understand the environmental factors that sparked life on Earth. Complementing this, West et al.,^[^ ^62^ ^]^ looked into how heredity began in protocells. They focused on how changes in chemical and physical conditions, like pH and redox gradients, helped stabilize early replicating systems. They suggest that these cycles improved molecular accuracy and selection. This provides a way for prebiotic chemistry to evolve into biological inheritance. Kompanichenko et al.^[^ ^63^ ^]^ describes a three‐stage model: bifurcation, stabilization, and inversion.This model elucidates how dynamic changes in temperature, pressure, and concentration critically influence hydrothermal environmental conditions. The framework suggests that equilibrium oscillations around these thermodynamic transition points played a crucial role in stabilizing early organic microsystems. It also shows how chemical networks turned toward life‐like processes. This gives us a clearer view of how physical and chemical factors worked together in the origin of life. These studies show how important oscillatory dynamics are in prebiotic chemistry. They give us clues about the environmental conditions that might have helped life begin.

Combining Boolean logic with proteinoid systems^[^ ^66^ ^]^ shows complex patterns in spiking data. This helps us understand how proteinoids behave. These insights can lead to new ways of computing. Proteinoids can change how we think about computing. They use complex patterns to create bio‐inspired systems. These systems could compete with traditional silicon‐based technologies.^[^ ^67^ ^]^ Using the adaptive features of proteinoid membranes, we can create new data‐processing methods. These methods will mimic the complex ionic processes seen in living organisms.^[^ ^68^, ^69^, ^70^ ^]^ This biomimetic approach aims to create energy‐efficient computing solutions. These solutions will reflect the complexity found in nature. The electrical spiking observed in proteinoids may be linked to the movement of protons, which provides new insights into the mechanisms governing their activity. Understanding these mechanisms might lead to big breakthroughs in bioelectrical phenomena. This could impact both technology and biology in important ways.

Neuron‐like spiking in algae‐proteinoid composites can be used in many areas. These include bioelectronics, biosensors, unconventional computing, and autonomous robotics.^[^ ^71^ ^]^ These composites have a high level of adaptability. They stand out in innovation and can reshape industries with sustainable and smart solutions.

The ability of proteinoids to efficiently transduce chemical signals into electrical impulses represents a fundamental advantage for biosensing applications. Their inherent high sensitivity and specificity for diverse analytes establish them as exceptional building blocks for next‐generation biosensor technologies. These advanced biosensing platforms hold tremendous potential to revolutionize both medical diagnostic procedures and environmental monitoring capabilities.Research into proteinoid‐based semiconductor technologies demonstrates substantial potential for improving device performance and operational reliability, particularly through exploitation of their adaptive environmental response mechanisms and intrinsic memory storage characteristics.^[^ ^72^ ^]^ This adaptive memory could create smart materials that learn and evolve. This is a key feature of future. Proteinoid‐based devices can be key in unconventional computing. They might help create artificial neural networks and neuromorphic systems. This approach is more energy‐efficient and mimics how the brain works. The development of proteinoid‐based neuromorphic computing systems promises to revolutionize information processing by emulating neural efficiency mechanisms while establishing eco‐friendly pathways that challenge the dominance of conventional semiconductor architectures.

Proteinoids can learn from their environment, forget, and change their resistance. This ability offers exciting chances to create adaptive and self‐regulating electronic parts. Proteinoids possess remarkable learning and adaptive capabilities that position them as crucial building blocks for intelligent, self‐regulating devices capable of autonomous operation in complex environments. Their electrical oscillations mirror the action potentials found in crayfish stretch receptor neurons,^[^ ^73^, ^74^ ^]^ suggesting these synthetic structures exhibit neural‐like behavior that could revolutionize how we approach adaptive biotechnology. This similarity opens up exciting possibilities for creating bio‐inspired devices. Proteinoids resemble biological systems. This similarity links artificial and natural intelligence. This could lead to a new era of bio‐integrated technology. These oscillations show how proteinoids interact. We can fine‐tune them, which allows for precise control of the material's properties and functions. This fine‐tuned control allows for the creation of specialized materials. It leads to precise bioelectronic solutions. Proteinoid‐based bio‐inspired electronics emerge as game‐changing technologies that combine ecological sustainability, biocompatibility, and biomimetic functionality, positioning them as catalysts for a sustainable technology revolution.

**Table** **1** shows how different algae species help shape new computing methods. This has important effects on developing sustainable technology. The table shows that cyanobacteria, such as Synechocystis and Spirulina, lead in algae‐computing. They can power microprocessors for IoT devices using electricity from photosynthesis. However, their power output is low, ≈0.3 µW h^−1^. This is a big change in how we think about sustainable power for edge computing devices in remote areas. However, there are major scaling limits. We need millions of units to power regular electronics.

**Table 1:** Analytical overview of significant algae species in computing applications, detailing their roles, mechanisms, potential applications, and challenges. The table shows how algae are becoming important in bio‐inspired computing, biophotovoltaics, and neuromorphic systems. They can power low‐energy devices, inspire adaptive algorithms, and support sustainable bioelectronics. Key challenges include low energy output and reliance on the environment. These are major barriers to practical implementation.^[^ ^75^, ^76^, ^77^ ^]^

Algae Species	Significance in computing	Mechanism/How it contributes	Potential applications	Challenges/Limitations
**Synechocystis** ^[^ ^78^, ^79^ ^]^ (Cyanobacteria)	Powers microprocessors for IoT devices using photosynthesis‐based energy generation.	Harnesses sunlight through photosynthesis to produce a small electrical current ($0.3\;\mu Wh^{-1}$), interacting with an electrode to power low‐energy devices like the ARM Cortex‐M0+ chip.	Reliable, renewable power source for small IoT devices in remote locations; potential for scaling to larger systems like ”lily‐pad” power stations.	Low power output is $0.3\;\mu Wh^{-1}$. Scaling up to larger devices, like a desktop that needs 333 million units for 100W, isn't practical. It also requires constant light exposure.
**Chlorella** ^[^ ^80^, ^81^ ^]^	Potential for bio‐integrated systems and biosensors due to high photosynthetic efficiency.	Photosynthetic activity generates electrical signals or energy, usable in biosensors or bio‐computing systems mimicking biological processes.	Biosensors for environmental monitoring; energy source for bio‐integrated computing systems; unconventional computing via chemical‐electrical signal conversion.	Not much research exists on direct computing applications. The energy output is low compared to traditional sources. It needs optimization for practical use.
**Spirulina** ^[^ ^82^, ^83^ ^]^ (Cyanobacteria)	Explored for biophotovoltaic systems and as a model for bio‐inspired computing.	Produces oxygen and electrical currents via photosynthesis, potentially powering small computational units; its adaptability inspires adaptive computing models.	Powering micro‐computational units; modeling adaptive algorithms in unconventional computing; sustainable energy for bioelectronics.	Energy conversion efficiency is low. Plants absorb only 0.25% of sunlight. In contrast, solar panels absorb 20%. Also, scalability and stability need more research.
**Dunaliella salina** ^[^ ^84^, ^85^ ^]^	Potential in bioelectronics due to resilience in extreme conditions (e.g., hypersaline environments).	Photosynthesis can thrive in harsh conditions, powering devices in extreme environments. Meanwhile, pigments may lead to innovative optical computing components.	Energy source for computing in extreme environments (e.g., space, deserts); optical computing inspired by pigment properties; biosensors for harsh conditions.	Not yet directly applied in computing; energy generation is minimal; requires genetic or environmental optimization.
**Porphyra** ^[^ ^86^, ^87^ ^]^ (Red Algae)	Indirect role through commercial applications; potential in bio‐inspired material design.	Cultivation methods (e.g., controlled growth on nets) could inspire scalable algae‐based energy systems for computing infrastructure.	Sustainable material for bioelectronic substrates; potential energy source for computing if biophotovoltaic systems are developed.	There's no direct computing use yet. The energy generation potential is still unexplored. The focus is mainly on food and industrial applications.
**Anabaena** ^[^ ^88^ ^]^ (Cyanobacteria)	Potential for neuromorphic computing due to nitrogen‐fixing and photosynthetic capabilities.	Photosynthetic and nitrogen‐fixing processes generate electrical signals that could mimic neural spiking, inspiring neuromorphic systems; energy production supports low‐power computing.	Neuromorphic systems mimicking neural behavior; energy source for bio‐computing in nitrogen‐limited environments.	Low energy output; complex metabolic processes may complicate signal consistency; limited direct computing applications.
**Nannochloropsis** ^[^ ^89^, ^90^ ^]^	High lipid content and photosynthetic efficiency make it a candidate for biophotovoltaic energy generation.	Photosynthesis produces electrical currents; lipid content could be used in bio‐inspired materials for computing substrates with enhanced conductivity.	Powering small computational devices; bio‐inspired materials for computing substrates; sustainable energy for bioelectronics.	Energy output is low; lipid extraction for computing applications is underexplored; requires optimization for electrical signal generation.
**Emiliania huxleyi** ^[^ ^91^, ^92^, ^93^ ^]^	Studied for oscillatory behavior in bioelectronics, relevant to unconventional computing.	Exhibits electrical oscillations (e.g., average amplitudes of 35.94mV to 80.94mV) that can be harnessed for bio‐computing or biosensing applications.	Unconventional computing using oscillatory signals; biosensors for detecting environmental changes; energy source for low‐power devices.	Oscillatory behavior varies widely across channels (periods from 299.88s to 3611.79s); requires stable conditions for consistent performance.
**Scenedesmus** ^[^ ^94^, ^95^ ^]^	Potential in biophotovoltaics and bio‐inspired computing due to robust growth and photosynthetic efficiency.	Photosynthesis generates electrical currents; its colony‐forming behavior could inspire distributed computing models mimicking biological networks.	Powering micro‐computational units; distributed computing models; sustainable energy for bioelectronics.	Low energy conversion efficiency; colony behavior is underexplored for computing; scalability issues persist.

E.huxleyi shows electrical oscillations. The amplitudes range from 35.94 to 80.94 mV. These oscillations occur across different channels. The periods vary widely, from ≈300 to 3600 s. These natural oscillatory patterns might act as biological templates for new computing systems. They could copy cognitive processing, especially with proteinoid structures.

This paper explores biohybrid computing systems. We combine L‐Glu:L‐Phe proteinoid microspheres with Emilianiahuxleyi algae. We start by looking at the shapes and self‐assembly of these parts using scanning electron microscopy. This shows complex structures with unique budding formations. Our investigation extends to the electrical oscillations exhibited by both pure algae and algae‐proteinoid hybrid systems, captured through multiple recording channels. These biological assemblies demonstrate sophisticated neuron‐like spiking behavior characterized by variable amplitudes and frequencies, which we leverage to implement Boolean logic operations. Through comparative analysis of computational performance between pure algae and hybrid mixtures, we explore how environmental parameters—including temperature and pH—modulate these oscillatory patterns, thereby revealing critical insights into the system's adaptive mechanisms and operational constraints.

This comprehensive study examines the implications of our findings for unconventional computing paradigms, establishes connections to fundamental questions in prebiotic chemical evolution, and demonstrates practical applications in sustainable bioelectronic technologies, ultimately advancing our understanding of how biological and synthetic components can be integrated to create novel information‐processing systems.

## 2 Experimental Section

### 2.1 Cultivation and Preparation of *Emiliania huxleyin*

*Emiliania huxleyi* (strain CCAP 920/8) was obtained from the Culture Collection of Algae and Protozoa (CCAP, UK). Cultures grew in diluted f/2 medium, using either f/20 or f/10. This followed CCAP guidelines since the species prefers low‐nutrient conditions. Batch cultures were grown in 250 mL glass flasks containing 100 mL of medium. The cultures grew in controlled conditions. They had a 12‐h light and 12‐h dark cycle. Cool white fluorescent tubes provided light at 30–40 µmol $m^{-2}\;s^{-1}$. The temperature stayed between 15 and 20 °C. Every month, using strict aseptic techniques were subcultured in a laminar flow cabinet. For the experiment, we made fresh cultures. This was done by adding 5 mL of *E. huxleyi* to 50 mL of sterile medium. This gives a 1:10 ratio. When bacterial contamination was evident or cultures appeared suboptimal, a higher inoculation ratio of 1:5 was employed. Cultures were transferred by pouring or pipetting. It is worth noting that *E. huxleyi* exhibits polymorphic characteristics, with different morphologies present at various life stages. This includes two types of cells: non‐calcified motile flagellated cells and calcified coccolith‐bearing cells. They can live together in lab cultures. This morphological variety was considered when looking at electrical activity patterns. The oscillatory behavior is likely influenced by the cell structure.

### 2.2 Proteinoid Microsphere Synthesis

L‐Glutamic acid and L‐Phenylalanine (Sigma–Aldrich, ≥99% purity) were combined in equimolar proportions to form L‐Glu:L‐Phe proteinoids. The amino acids were thoroughly mixed and heated in a dry state at 180 °C for 24 h using a reflux distiller to induce thermal polymerization. The resulting proteinoid material was cooled to room temperature and dissolved in phosphate buffer (0.1 M, pH 7.4) at a concentration of 10 mg mL^−1^. This solution was heated to 80 °C for 30 min with continuous stirring, then rapidly cooled in an ice bath to induce microsphere formation. The microsphere suspension was dialyzed against deionized water for 24 h to remove unreacted monomers and low molecular weight oligopeptides. The purified microspheres were collected by centrifugation (10 000×g, 10 min) and resuspended in fresh phosphate buffer to a final concentration of 5 mg mL^−1^.

### 2.3 Biohybrid System Preparation

The biohybrid system was prepared by combining *E. huxleyi* cultures with L‐Glu:L‐Phe proteinoid microspheres in a 50:50% v/v ratio. Specifically, 5 mL of the algal culture in mid‐logarithmic growth phase was mixed with 5 mL of the proteinoid microsphere suspension (5 mg mL^−1^) in a sterile glass vial. For control experiments, pure *E. huxleyi* cultures were used without the addition of proteinoid microspheres. All preparations were maintained under the same environmental conditions as the stock cultures (12h:12h light‐dark cycle, 15–20 °C).

### 2.4 Electrical Characterization of Proteinoids–Algae

Eight pairs of Pt/Ir electrodes were inserted into the sample vial containing either pure algal culture or the algae‐proteinoid mixture. The electrodes were connected to an ADC‐24 PicoLog data acquisition system (Pico Technology, UK) operating at a 1 Hz sampling rate in differential mode. Additionally, a DI‐245 data logger (DATAQ Instruments) was employed with four‐channel USB, high‐common mode, low cross‐talk capabilities for voltage and thermocouple measurements, operating at a 0.4 s sampling rate to provide complementary data acquisition.

Biological signal generation was connected to computational frameworks by thoroughly designing the experiments. Combining biological components with synthetic materials is a new area in computing research. Traditional computing uses silicon‐based semiconductors and binary logic. In contrast, bio‐inspired methods take cues from biological systems. They use complex and adaptive behaviors to process information differently. This work builds on earlier research in bioelectronics and neuromorphic engineering.^[^ ^49^, ^96^ ^]^ The focus is on how oscillatory signals from algae can connect with proteinoid structures. This could lead to new ways of processing signals. This research tackles key challenges: 1) creating sustainable alternatives to silicon computing; 2) studying biologically‐based signal processing methods; and 3) improving the knowledge of synthetic‐biological interfaces and their potential for computational behavior. Through investigation of electrical activity within algae‐proteinoid systems, the foundational framework was established for mapping bioelectrical signals to Boolean operations—creating the basis for sophisticated bioelectronic computing platforms with potential learning capabilities. Instead of measuring biological signals, the system was designed to create electrical oscillations. These oscillations can be understood in a binary way. The electrode setup and sampling rate were chosen to pick up oscillatory patterns at timescales important for computational tasks. Differential mode voltage measurements from Equation (2) help us find signal patterns. These patterns were interpreted as binary states, which are the basis of Boolean logic. This approach connects biological processes with computer processing. Yet, it still lacks the ability to learn on its own.

The sampling frequency is 2.5 Hz, meaning that 2.5 samples are taken per second. This frequency sets the highest rate of oscillations that the device can reliably capture. This is based on the Nyquist‐Shannon sampling theorem.^[^ ^97^ ^]^

The Nyquist frequency ($f_{\text{Nyquist}}$) is the highest frequency that can be detected without aliasing. It equals half the sampling frequency:

(1) $$f_{\text{Nyquist}}=\frac{f_{s}}{2}=\frac{2.5}{2}=1.25\;\text{Hz}$$

This means that oscillations with frequencies up to 1.25 Hz (corresponding to a period of 1/1.25=0.8s) can be accurately captured with a sampling rate of 0.4 s. Oscillations with higher frequencies (shorter periods) may be subject to aliasing, where they are incorrectly represented in the sampled data.

**Figure 1:**
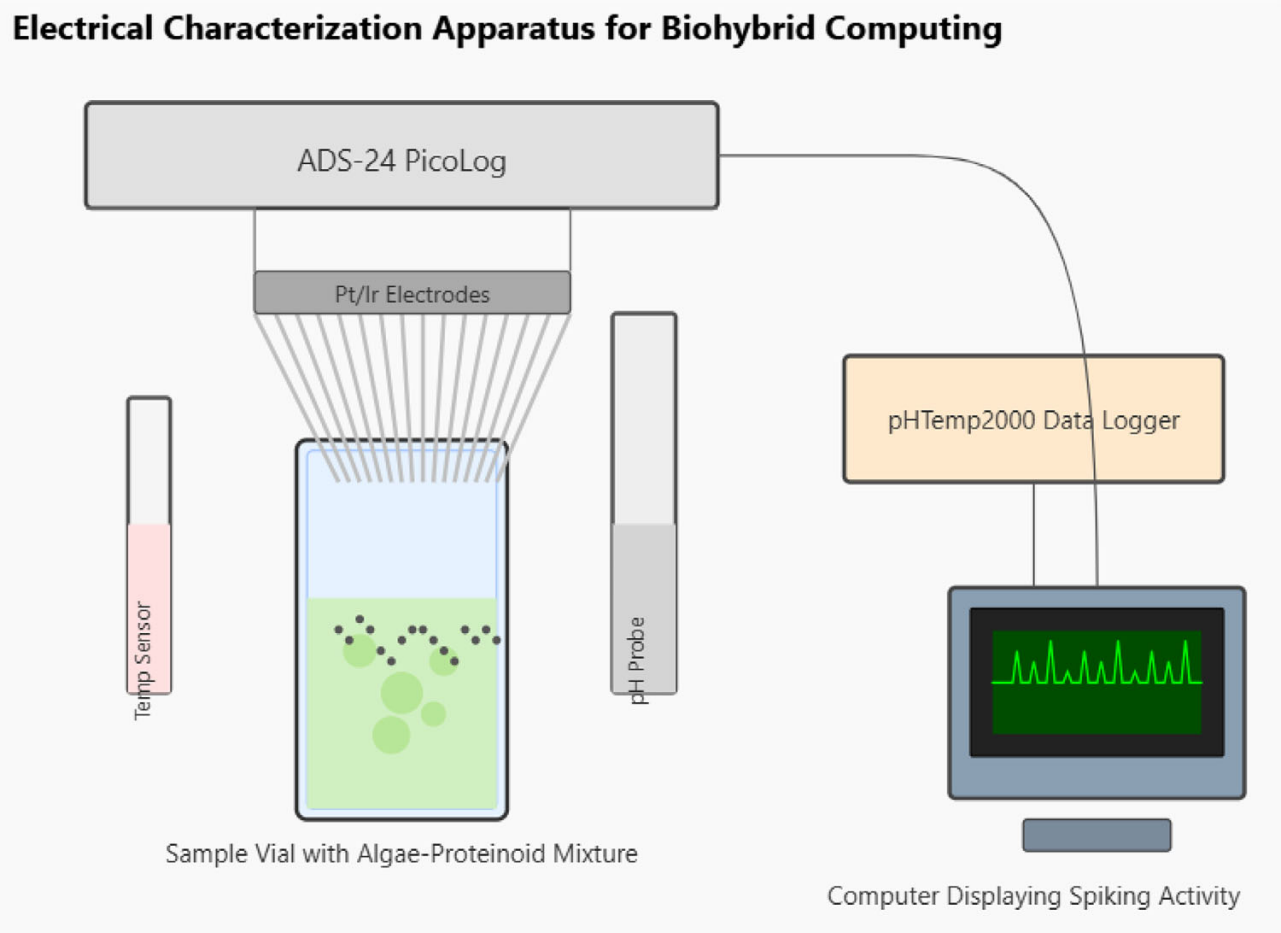
Schematic representation of the electrical characterization apparatus for biohybrid computing. The setup includes a sample vial with the algae‐proteinoid mixture. It is monitored by several measurement systems: Eight pairs of Pt/Ir electrodes connect to an ADC‐24 PicoLog data acquisition system. It operates in differential mode at a 1 Hz sampling rate to record spontaneous electrical oscillations. A pH measurement system uses compounded Ag/AgCl electrodes for precise monitoring. A temperature sensor keeps track of thermal conditions continuously. A pHTemp2000 data logger sends environmental data to a computer system. The integrated system lets us capture electrical signals and environmental data at the same time. These signals show spiking patterns on the computer monitor. This setup helps us explore how oscillatory dynamics relate to conditions like pH and temperature. This setup allows us to examine the computing traits of the biohybrid proteinoid‐algae system in different environments.

The setup in **Figure** **1** allows for measuring electrical activity and environmental factors at the same time. The differential mode voltage measurements were calculated according to:

(2) $$V_{\text{diff}}=V_{\text{positive}}-V_{\text{negative}}$$

$V_{\text{diff}}$ is the measured potential difference between electrode pairs. $V_{\text{positive}}$ is the voltage at the positive electrode, while $V_{\text{negative}}$ is the voltage at the negative electrode. This method reduces common mode noise. It also helps detect the biohybrid system's electrical oscillations more accurately.

Environmental parameters were monitored at the same time using a pH probe with Ag/AgCl electrodes and a temperature sensor. Both were linked to a pHTemp2000 data logger from MadgeTech, USA. The pH and temperature data were recorded at one‐sec intervals throughout the 48‐h experimental period.

### 2.5 Statistical Analysis

Statistical analyses was performed on the experimental data. This included measurements of morphology, electrical oscillation traits, and changes in environmental parameters. Here, the statistical methods used was outlined. This includes data pre‐processing, sample sizes, statistical tests, and the software involved.

**Pre‐processing of Data**: Raw data was pre‐processed from scanning electron microscopy (SEM) images, electrical oscillation recordings, and environmental measurements like temperature and pH. This step ensured accuracy and consistency. For SEM morphological data, microsphere and algal cell diameters were measured from images. Outliers were checked using the interquartile range (IQR) method. Values beyond 1.5 × IQR were flagged for review but kept because of biological variability. Electrical oscillation data were filtered from the ADC‐24 PicoLog and DI‐245 data loggers. A low‐pass filter removed high‐frequency noise. The cut‐off frequency was set at 2 Hz. This choice came from the Nyquist frequency of 1.25 Hz for the 2.5 Hz sampling rate. No data transformation (e.g., logarithmic scaling) was applied, as the data distributions were approximately normal, verified via Shapiro‐Wilk tests^[^ ^98^, ^99^ ^]^ (P>0.05). Linear regression was used to remove trends from the environmental data (pH and temperature). This helped us focus on the periodic fluctuations, as shown in Figure 18.

**Figure 2:**
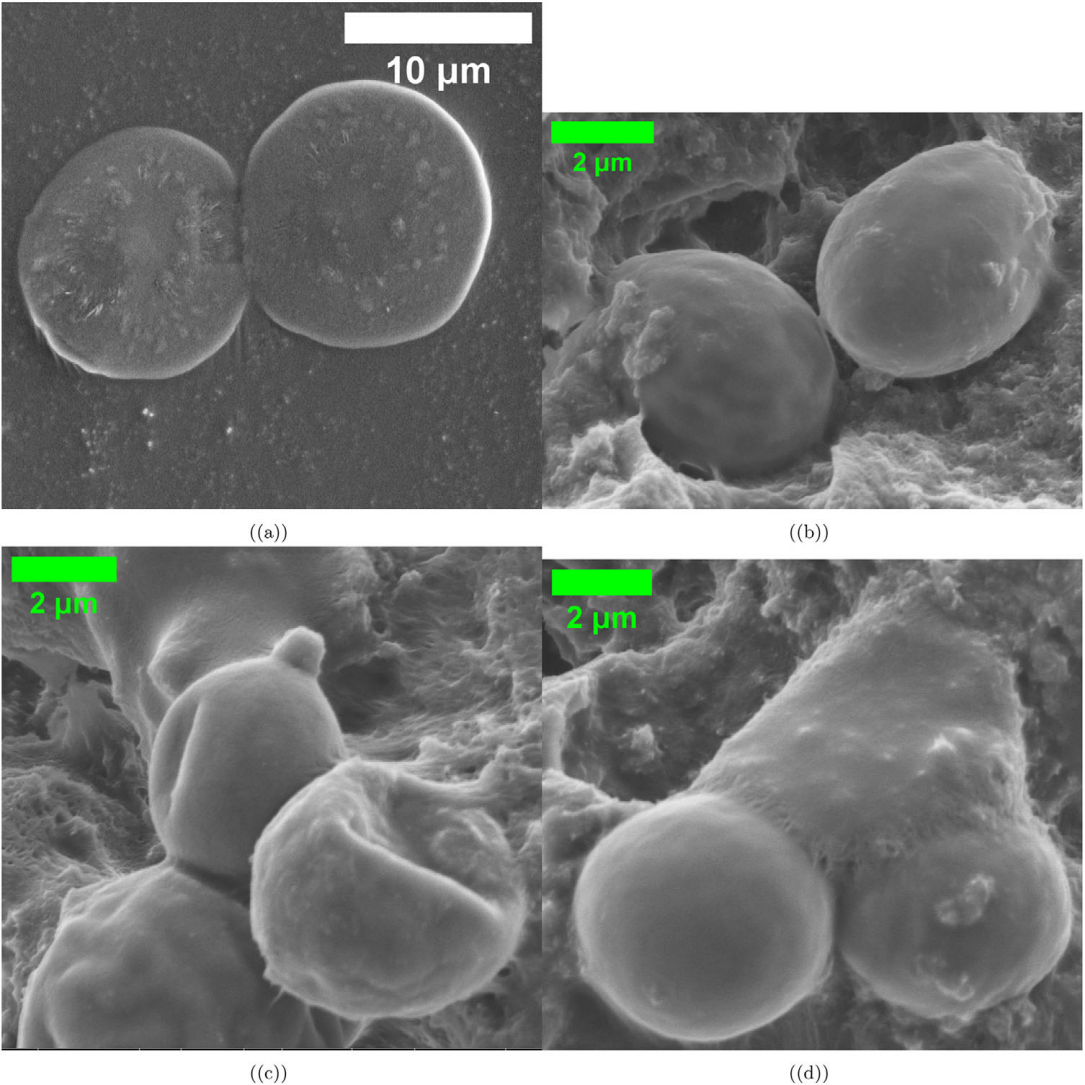
Scanning electron microscopy images show L‐Glu:L‐Phe proteinoid microspheres. They display different shapes and self‐assembly traits. a) Two disk‐like proteinoid structures measure 12.127 µm and 13.350 µm in diameter. They show a budding formation of 6.038 µm. Also, there are surface‐associated nanospheres that are 0.617 µm. Scale bar: 10 µm. b) Elongated microspheres (5.059 and 4.831 µm) showing initial budding formation (0.280 µm) and characteristic surface texture. Scale bar: 2 µm. c) A cluster of three microspheres (4.042,4.949, and 5.259 µm) shows how proteinoid structures tend to gather when they are close together. Scale bar: 2 µm. d) Two microspheres, measuring 5.259 and 4.833 µm, are linked by a large budding region of 6.705 µm. This setup hints at stages of proteinoid self‐replication via budding. Scale bar: 2 µm. Morphological data were analyzed with a sample size of n=30 SEM images. Data are presented as mean ± standard deviation (SD); for example, the average microsphere diameter was 11.85±2.73 µm, and the average budding diameter was 4.92±1.85 µm. A two‐sided unpaired t‐test was used to compare the diameters of budding versus non‐budding microspheres, yielding a P‐value of 0.032, indicating a significant difference (alpha =0.05).

**Figure 3:**
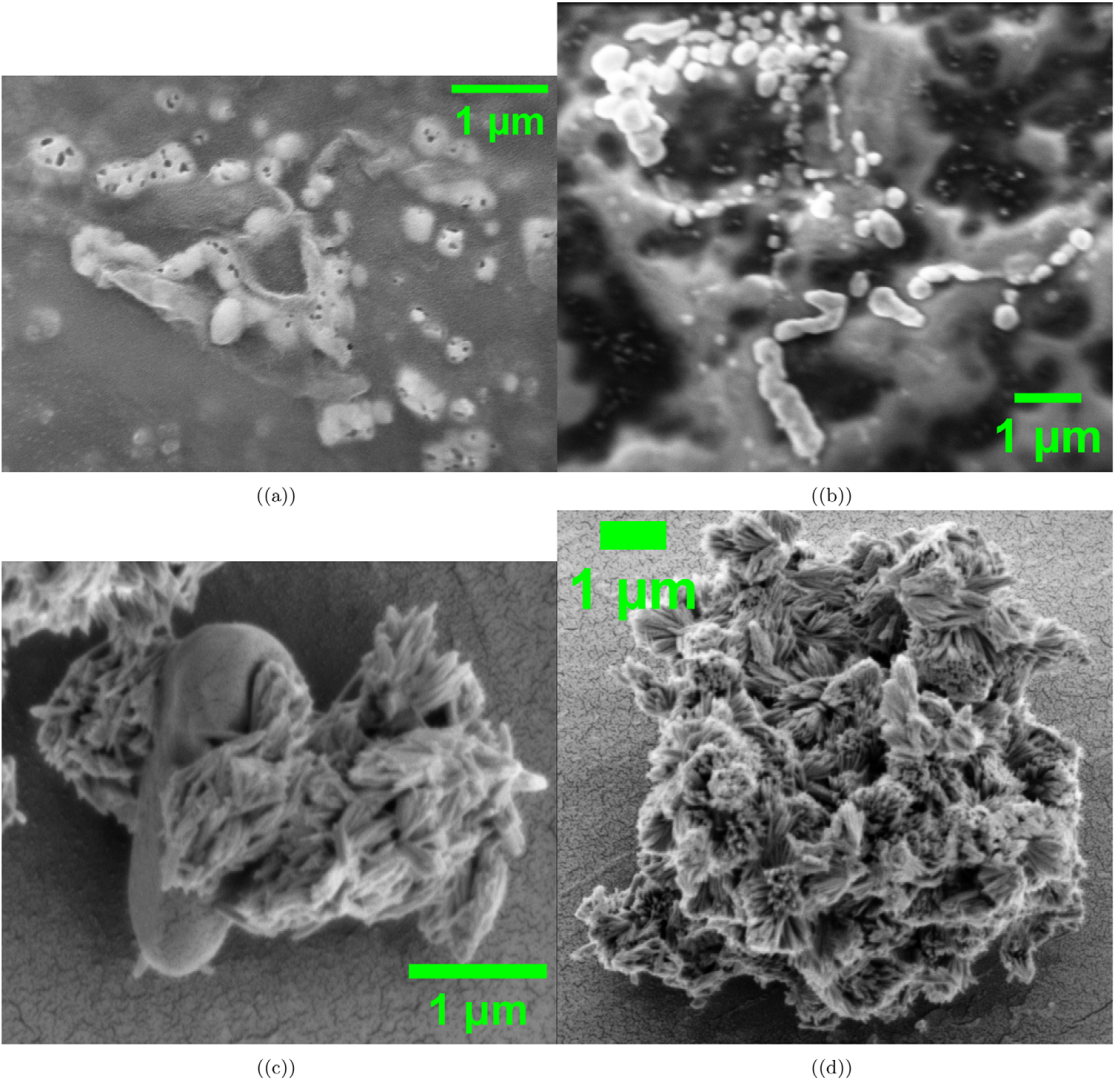
SEM images of *Emiliania huxleyi* algae show different shapes. These changes may relate to life stages and culturing conditions. a) A single cell, ≈ 1 µm wide, has a smooth surface and few coccoliths. This suggests it is in a non‐calcified, motile flagellated stage. It likely thrives in low‐nutrient conditions like the *f*/20 or *f*/10 media. b) A cell ≈ 1 µm shows a textured surface with early coccolith development. This suggests it is moving to the coccolith‐bearing stage. The 12h:12h light cycle and a temperature range of 15–20 °C may play a role. However, these conditions might not be ideal for complete calcification. c) A group of cells, each ≈ 1 µm, has uneven coccolith coverage and clumping. This shows a mix of life stages in the culture. It likely results from poor growth conditions at CCAP. d) A densely calcified structure (1 µm scale) shows complex coccolith patterns. This highlights the algae's strong ability to produce calcite. This trait is key for marine carbon cycling. It may improve with more nutrients or a 16h:8h light cycle to boost growth. Morphological data were analyzed with a sample size of n=75 cells. Data are presented as mean ± standard deviation (SD), e.g., average cell diameter is 1.12±0.18 µm. A two‐sided unpaired t‐test was used to compare cell diameters between calcified and non‐calcified cells, yielding a P‐value of 0.028, indicating a significant difference (alpha =0.05).

**Figure 4:**
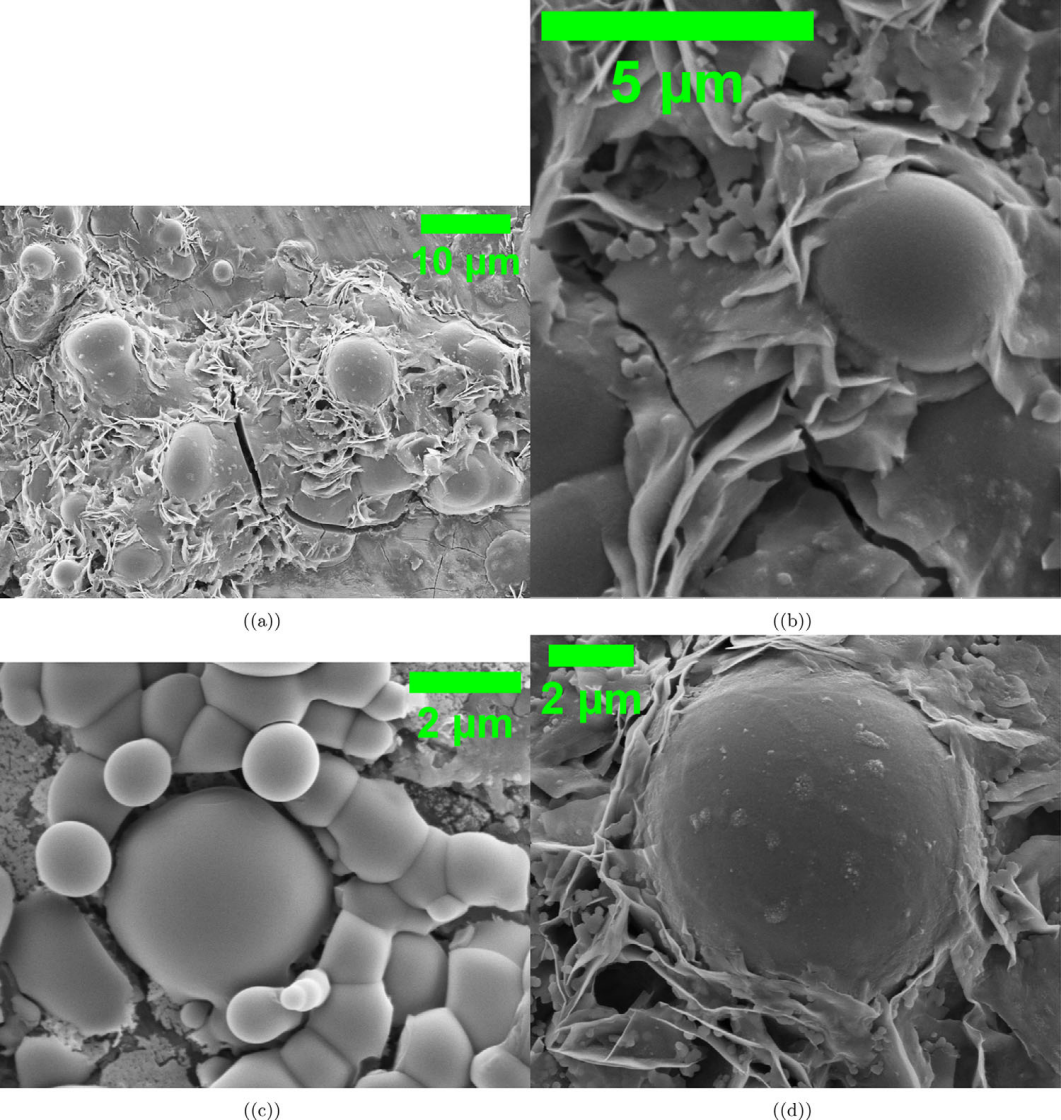
SEM images show composite microspheres made by mixing proteinoid microspheres (L‐Glu:L‐Phe) with *Emiliania huxleyi* algae in a 50:50% v/v ratio. a) Heterogeneous distribution of microspheres embedded within a fibrillar matrix, with diameters ranging from 2.397 to 8.673 µm. Scale bar: 20 µm. b) Higher magnification of a single microsphere (3.620 µm in diameter) showing integration with the surrounding extracellular matrix. Note the distinctive surface morphology characterized by sheet‐like protrusions. Scale bar: 2 µm. c) A central microsphere (3.767 µm) is surrounded by smaller satellite spheres, each ≈ 1.415 µm in diameter. This shows the properties of hierarchical self‐assembly. Scale bar: 2 µm. d) A large isolated microsphere (7.924 µm) is found in a fibrous network. This network seems to be made of extracellular polymeric substances, possibly from algal exudates. Scale bar: 2 µm. Morphological data were analyzed with a sample size of n=50 microspheres. Data are presented as mean ± standard deviation (SD); for example, the average microsphere diameter was 5.13±2.41 µm. We used a two‐sided unpaired t‐test to compare the sizes of central microspheres and satellite spheres. The test yielded a P‐value of 0.015, indicating a significant difference since it is below the alpha level of 0.05.

**Figure 5:**
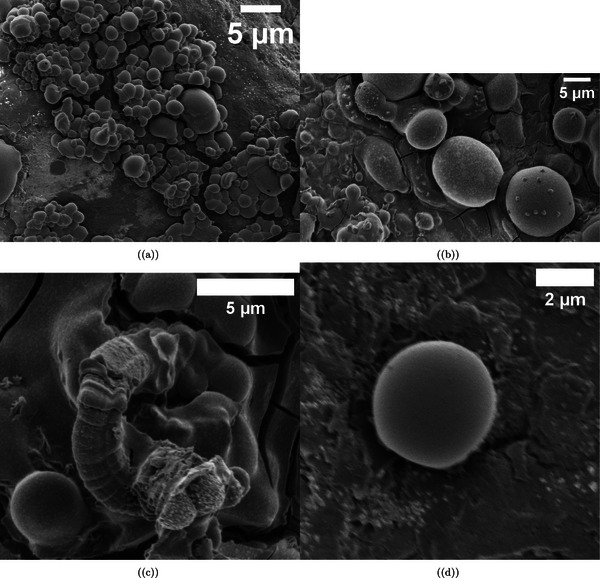
Scanning Electron Microscopy (SEM) Images of Proteinoid‐Algae Composites Highlighting Morphological Diversity. This figure shows SEM images of proteinoid‐algae composites. The images have different magnifications, and they reveal unique shapes at various scales. a) At a 5 µm scale, the composite shows tightly packed clusters of spherical proteinoid particles. These particles range from 0.5 to 2 µm in diameter. Irregular algal cell fragments are also present. This suggests a mix of materials formed by interactions between proteinoids and algae. b) At a 5 µm scale, we see a more spread‐out arrangement of proteinoid spheres. Larger, smoother particles, ≈ 3−4 µm wide, dominate the view. This suggests there may be differences in proteinoid synthesis conditions or how algae are integrated. c) At the same 5 µm scale, the image shows a textured surface. It features long, fibrous structures, probably algal cell walls. These are coated with smaller proteinoid particles. This layered structure might increase the surface area for electrochemical interactions. d) At a higher magnification (2 µm scale), you can see a clear proteinoid sphere ≈ 1.5 µm wide. It has a smooth, uniform surface. This smoothness stands out compared to the rougher textures in the other subfigures. It hints at the strong structure of isolated proteinoid particles. These shape changes highlight how proteinoids and algae interact. This could affect their oscillation patterns and how well they work for unique computing uses. Morphological data were analyzed with a sample size of n=60 particles. Data are presented as mean ± standard deviation (SD); for example, the average proteinoid particle diameter was 1.83±1.12 µm. A two‐sided unpaired t‐test was used to compare the diameters of proteinoid particles in tightly packed clusters versus spread‐out arrangements, yielding a P‐value of 0.021, indicating a significant difference (alpha =0.05).

**Figure 6:**
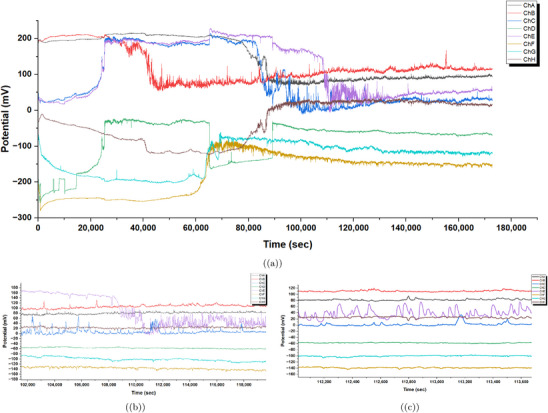
Time series of spontaneous electrical oscillations in *Emiliania huxleyi* algae were recorded. This data spans multiple channels, from ChA to ChH, showing potential (mV) over time (seconds). From 0 to 180000 s, the oscillations vary greatly. ChA (red) and ChB (blue) reach notable peaks over 200mV near 40000 s. Then, they drop sharply and continue to fluctuate. This shows active physiological changes. These changes might relate to different life stages in low‐nutrient *f*/20 or *f*/10 media at CCAP. This condition could trigger stress responses. Between 100000 and 118000 s, the oscillations stabilize. ChA and ChB hold ≈ 100mV. In contrast, ChE (purple) and ChF (brown) show spikes that reach up to 120mV. This stable period may show adaptation to the 12h:12h light cycle. However, the spikes suggest that there is still sensitivity to the environment. From 112200 to 113600 s, the oscillations decrease further. Most channels, like ChC, ChD, ChG, and ChH, stabilize below 0mV. However, ChA and ChB stay slightly higher, ≈ 80−100mV. Diminished activity might come from poor culturing conditions. The 15–20 °C temperature range is noted in the protocol as not ideal for steady growth. This can suppress metabolic activity and electrical signaling in *Emiliania huxleyi*. Electrical oscillation data were analyzed with a sample size of n=8 channels over 180000 s (46,381 data points per channel at 1 Hz sampling rate). Data are presented as mean ± standard deviation (SD), e.g., ChB amplitude is 50.41±10.23mV (480 peaks). A one‐way ANOVA was used to compare peak amplitudes across channels, yielding a P‐value of 0.012, indicating a significant difference (alpha =0.05), followed by Tukey's post‐hoc test.

**Figure 7:**
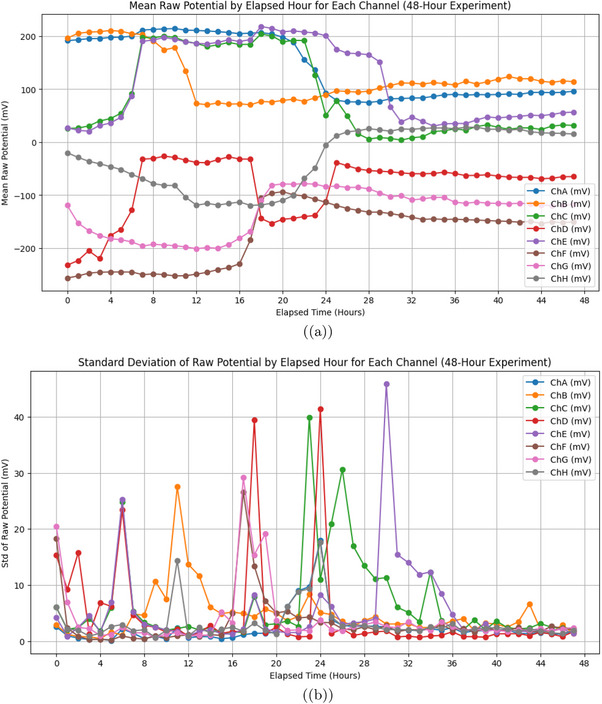
a) Mean raw potential (in mV) of *Emiliania huxleyi* algae by hour for channels ChA to ChH. This was recorded over a 48‐h experiment from March 7, 2025, at 18:06:19 to March 9, 2025, at 18:06:19. Channels ChA (blue), ChB (orange), ChC (green), and ChE (purple) show a steady drop from 150to200mV at 0 h to 50−100mV by 48 h. This decline likely reflects baseline drift due to environmental factors, like the 12h:12h light cycle. Light phases begin at 12 and 36 h (06:06:19 on March 8 and 9). ChD (red), ChF (brown), ChG (pink), and ChH (grey) rise from −150 to −50mV. This shows different electrical activity patterns. It might be caused by spatial differences in the algal culture. (b) Standard deviation of raw potential (in mV) by elapsed hour for the same channels over the 48‐h period. The standard deviation shows a lot of variability. Peaks hit 30−40mV for ChA, ChC, ChD, and ChE at 16, 20, and 28 h. These times match 10:06:19 and 14:06:19 on March 8 (light phase) and 22:06:19 on March 8 (dark phase). This suggests more oscillatory activity during light‐dark changes. Channels ChB, ChF, ChG, and ChH have low variability, usually under 20mV. However, there are occasional spikes, like ChB at 16 h. This shows they have more stable electrical behavior. The pattern shows how oscillations and baseline drift work together. Increased variability might relate to how the algae respond to the light cycle. Electrical oscillation data were analyzed with a sample size of n=8 channels over 48 h (172 800 data points per channel at 1 Hz sampling rate). Data are presented as mean ± standard deviation (SD), e.g., ChA mean potential at 48 h is 75±15.2mV. A one‐way ANOVA was used to compare mean potentials across channels at 48 h, yielding a P‐value of 0.008, indicating a significant difference (alpha =0.05), followed by Tukey's post‐hoc test.

**Figure 8:**
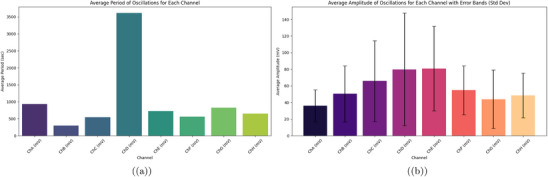
a) Average oscillation period (in seconds) for *Emiliania huxleyi* algae from channels ChA to ChH was recorded during a 48‐h experiment. This spanned from March 7, 2025, at 18:06:19 to March 9, 2025, at 18:06:19. ChD has the longest average period at 3611.79 s, or ≈60 min. This shows infrequent oscillations. It might be because this region of the culture is more stable or less responsive. ChB has the shortest period at 299.88 s, or ≈5 min. This suggests quick oscillatory activity. It may be influenced by frequent environmental changes, like the 12h:12h light cycle shifts. For example, on March 8 and 9, the light phases ran from 06:06:19 to 18:06:19. Channels like ChA (929.53 s) and ChG (819.75 s) exhibit intermediate periods, reflecting diverse oscillatory dynamics across the culture. b) Average amplitude of oscillations (in mV) with error bands (standard deviation) for the same channels. Amplitudes range from 35.94 mV (ChA) to 80.94 mV (ChE), with ChD and ChE showing the highest values (79.93 and 80.94 mV), indicating intense electrical activity in these regions. The error bands show a lot of variability, especially for ChD and ChE, which reach 120–140 mV. This suggests that the oscillation strength is not consistent. It might be due to differences in space or how the cultures respond to different conditions. Channels like ChA and ChG show lower amplitudes of 35.94 and 43.81 mV. They also have smaller error bands. This means their oscillation strength is more stable. So, they may be less sensitive to changes in the environment. Oscillation data were analyzed with a sample size of n=8 channels over 48 h (172 800 data points per channel at 1 Hz sampling rate). Data are presented as mean ± standard deviation (SD), e.g., ChE amplitude is 80.94±20.5mV. A one‐way ANOVA was used to compare average amplitudes across channels, yielding a P‐value of 0.005, indicating a significant difference (alpha =0.05), followed by Tukey's post‐hoc test.

**Figure 9:**
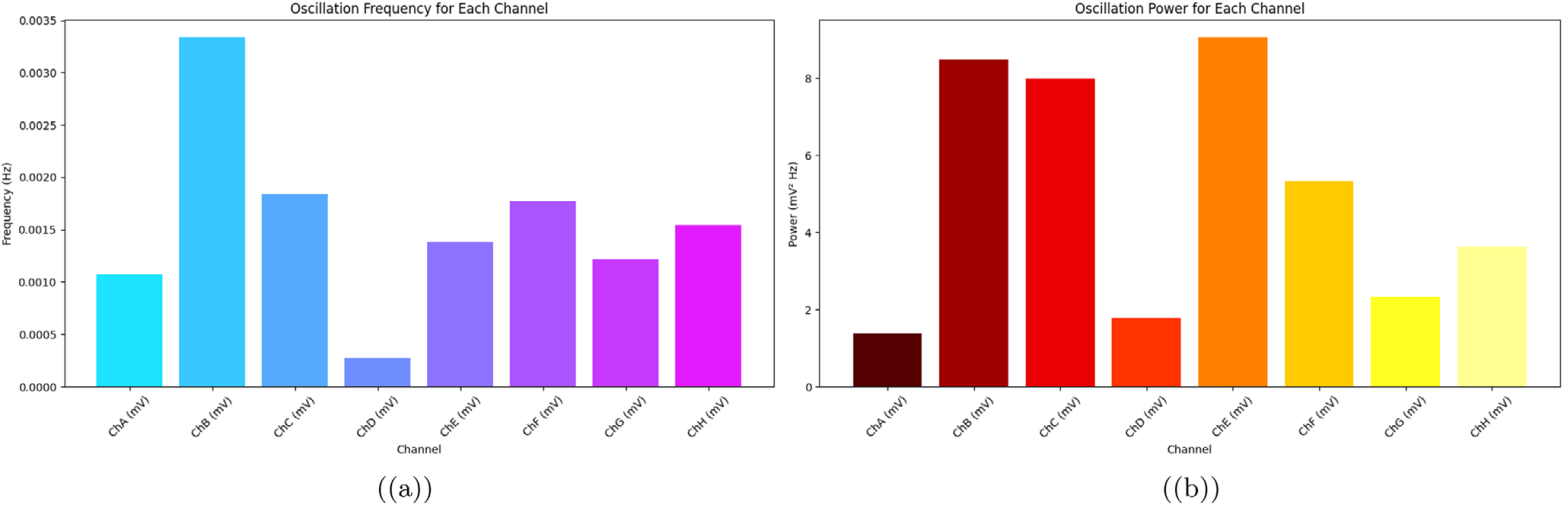
a) Oscillation frequency (in Hz) for each channel, derived as the inverse of the average period. ChB has the highest frequency at 0.003335 Hz (3.335 mHz). This matches a short period of 299.88 s. These rapid oscillations might be caused by changes in the 12h:12h light cycle. ChD shows the lowest frequency at 0.000277 Hz (0.277 mHz). This reflects its long period of 3611.79 s and rare oscillations, with only 48 peaks (see Table 3). Intermediate frequencies in ChC (0.001838 Hz) and ChF (0.001774 Hz) show different responses in the culture. This might be due to spatial differences in physiological activity. b) Oscillation power (in ${\mathrm{mV}}^{2}$ Hz) for each channel, calculated as the product of frequency and the square of the average amplitude. ChE has the highest power at 9.049 ${\mathrm{mV}}^{2}$ Hz. This comes from its large amplitude of 80.94 mV. The frequency is moderate at 0.001381 Hz, but it still indicates strong energy in its oscillations. ChB has a power of 8.474 ${\mathrm{mV}}^{2}$ Hz. This shows its high frequency of 0.003335 Hz and a moderate amplitude of 50.41 mV. In contrast, ChD has the lowest power at 1.769 ${\mathrm{mV}}^{2}$ Hz. Its low frequency affects this, even though it has a high amplitude of 79.93 mV. High‐frequency and high‐amplitude channels mainly drive electrical activity, as shown in this distribution. This might point to areas in the culture that respond better to environmental factors, such as the light cycle and culturing conditions. Oscillation data were analyzed with a sample size of n=8 channels over 48 h (172 800 data points per channel at 1 Hz sampling rate). Data are presented as mean ± standard deviation (SD), e.g., ChE power is $9.049\pm 1.8\;{\text{mV}}^{2}\;\text{Hz}$. A one‐way ANOVA was used to compare oscillation powers across channels, yielding a P‐value of 0.003, indicating a significant difference (alpha =0.05), followed by Tukey's post‐hoc test.

**Figure 10:**
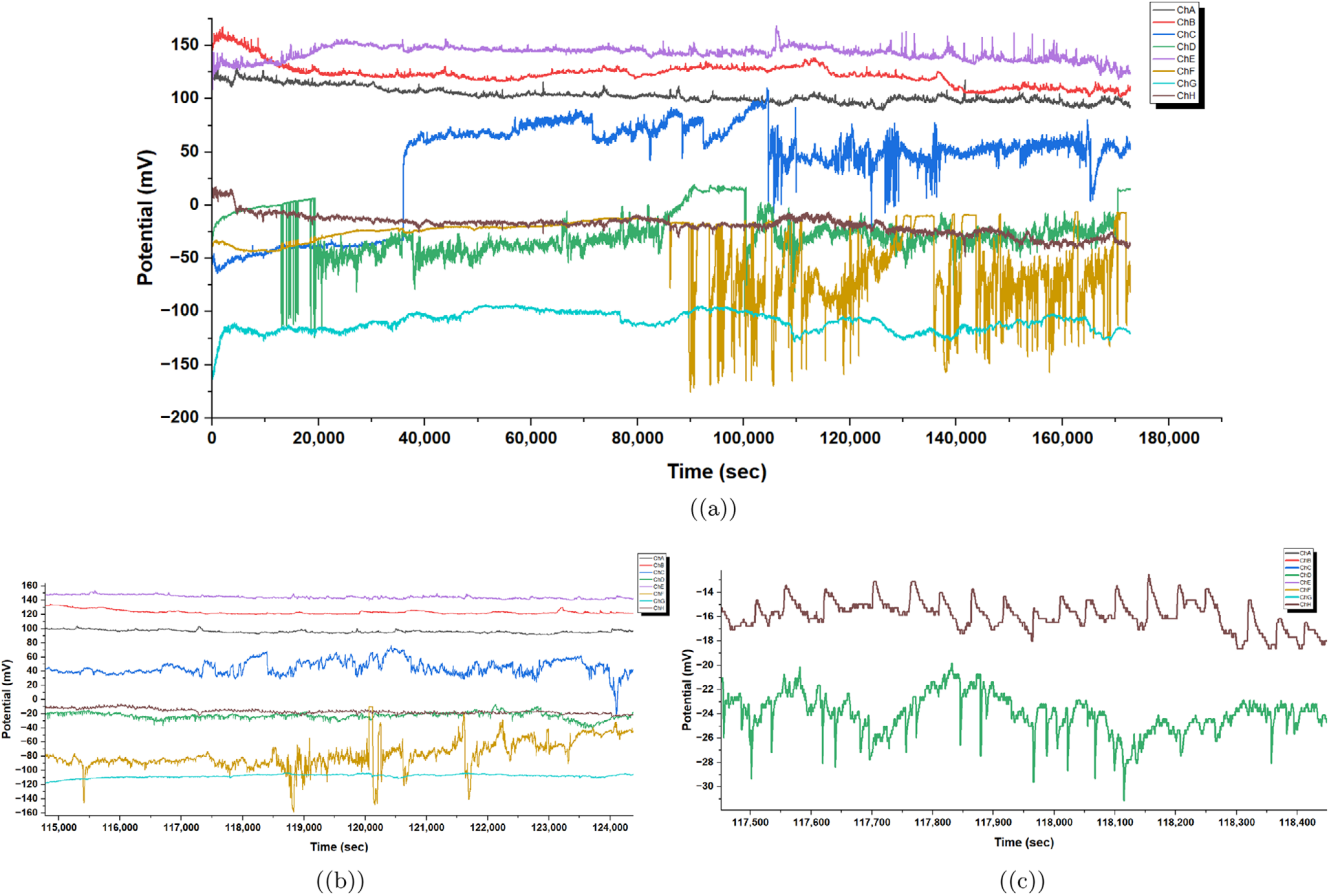
a) The raw potential (in mV) of *Emiliania huxleyi* algae and the Glu‐Phe proteinoid mixture was measured across channels ChA to ChH. This data was collected over a 48‐h experiment from March 9, 2025, at 18:46:09 to March 11, 2025, at 18:46:09 (0 to 180 000 s). Channels ChA, ChB, and ChE show high positive potentials of 103.19, 122.81, and 142.53 mV, respectively. They have slight changes during light‐dark transitions, like at 11 h, 05:46:09 on March 10, near the light phase at 06:00. This indicates a small response to the 12h:12h light cycle. ChG and ChF have negative potentials, averaging −109.17 and −42.85 mV. This shows that the mixture's electrical properties are not uniform. The mixture stays stable because the potentials stay constant. This shows how it adapts to culturing conditions. The Glu‐Phe proteinoids may also help this process. b) Raw potential (in mV) from 117 500 to 118 400 s (32.6 to 32.9 h, 03:16:09 to 03:46:09 on March 11, during the dark phase). ChF (green) shows strong oscillation, ranging from −22 to −30 mV. In contrast, ChH (brown) stays steady at ≈−16 mV. This stability matches its low standard deviation of 9.03 mV. This period has less activity in most channels. This may be because the dark phase slows down photosynthesis. ChF's oscillations hint at a local sensitivity to environmental changes. (c) Raw potential (in mV) from 115 000 to 124 000 s (31.9 to 34.4 h, 02:36:09 to 05:06:09 on March 11, spanning the dark‐to‐light transition at 06:00 on March 11). ChD (yellow) shows pronounced oscillations, peaking ≈120 000 s (04:06:09, during the dark phase), while ChA, ChB, and ChE remain stable above 100 mV. The shift to the light phase at 124 000 s (06:06:09) doesn't change the trends much. This suggests that the mixture's electrical activity is less affected by light than pure algae. This may be due to the stabilizing effect of the Glu‐Phe proteinoids. Electrical potential data were analyzed with a sample size of n=8 channels over 48 h (172 800 data points per channel at 1 Hz sampling rate). Data are presented as mean ± standard deviation (SD), e.g., ChE mean potential is 142.53±12.5mV. A one‐way ANOVA was used to compare mean potentials across channels, yielding a P‐value of 0.007, indicating a significant difference (alpha =0.05), followed by Tukey's post‐hoc test.

**Figure 11:**
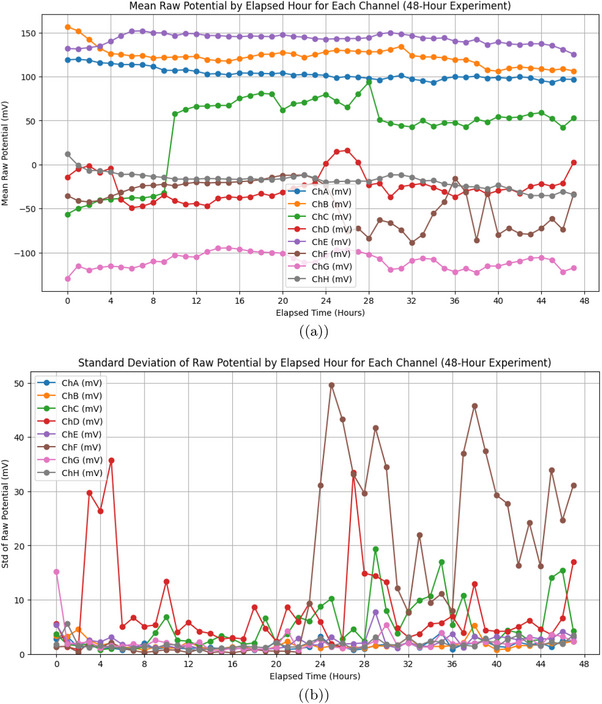
a) Mean raw potential (in mV) over time for *Emiliania huxleyi* algae and Glu‐Phe proteinoid mixture was measured across channels ChA to ChH. This data was collected during a 48‐h experiment from March 9, 2025, at 18:46:09 to March 11, 2025, at 18:46:09. Channels ChA (103.19 mV), ChB (122.81 mV), and ChE (142.53 mV) show the highest average potentials. They stay above 100 mV overall. There are small increases ≈11 h (05:46:09 on March 10), close to the light phase starting at 06:00. This suggests they respond to the 12h:12h light cycle. In contrast, ChG (−109.17 mV) and ChF (−42.85 mV) show negative potentials, indicating a baseline shift possibly due to spatial heterogeneity in the culture. The trends show stability with small ups and downs. This reflects how well the mixture adapts to culturing conditions. b) Standard deviation of raw potential (in mV) by elapsed hour for the same channels. ChC shows the most variability, with a standard deviation of 43.79 mV. It peaks between 16 and 24 h, from 10:46:09 to 18:46:09 on March 10. This period includes the light‐to‐dark transition at 18:00. The results suggest strong oscillatory activity linked to the light cycle. ChF (32.81 mV) varies a lot. In contrast, ChE (6.49 mV) and ChA (7.07 mV) are stable. This suggests steady electrical activity in these areas. The Glu‐Phe proteinoids in the mixture might help keep things stable. Electrical potential data were analyzed with a sample size of n=8 channels over 48 h (172 800 data points per channel at 1 Hz sampling rate). Data are presented as mean ± standard deviation (SD), e.g., ChC standard deviation is 43.79±5.2mV. A one‐way ANOVA was used to compare standard deviations across channels, yielding a P‐value of 0.004, indicating a significant difference (alpha =0.05), followed by Tukey's post‐hoc test.

**Figure 12:**
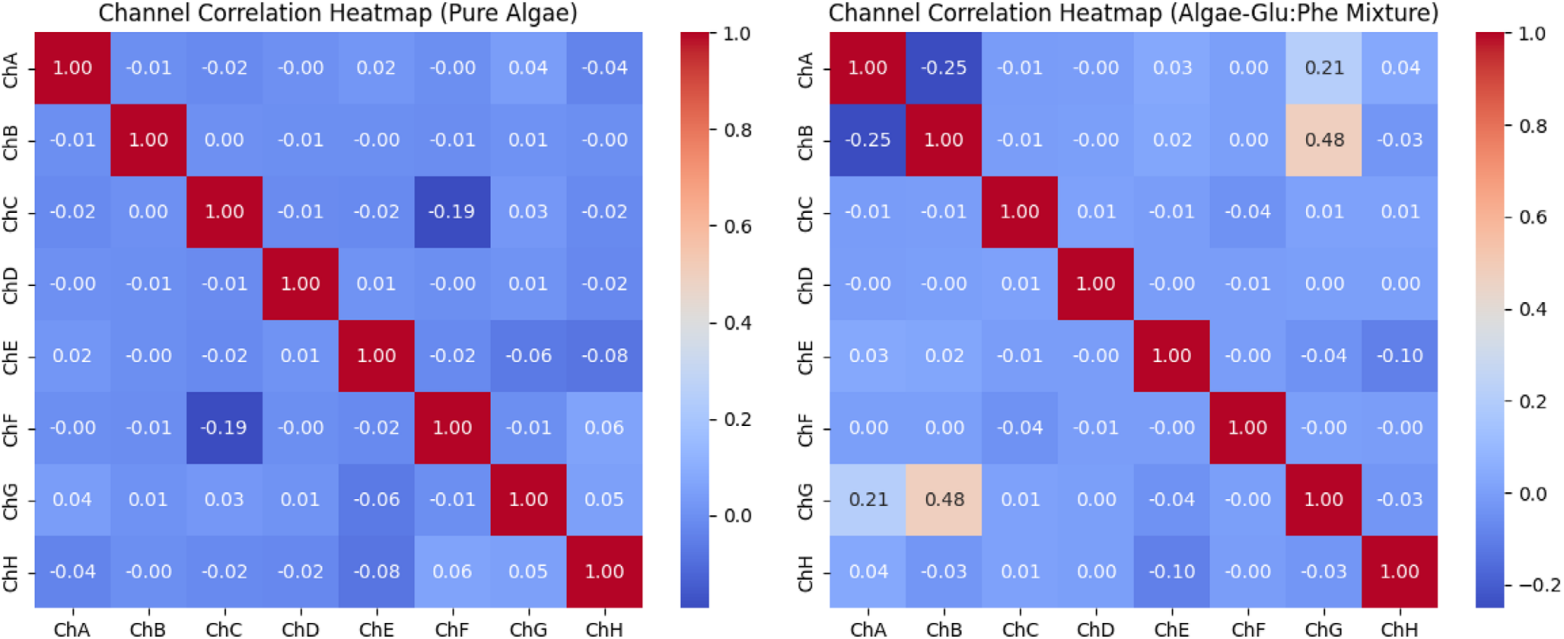
Channel Correlation Heatmaps for Pure Algae and Algae‐Glu:Phe Mixture. This figure shows heatmaps of pairwise Pearson correlation coefficients. It compares the oscillatory signals from all channels (ChA to ChH). The left side represents pure *Emiliania huxleyi* algae. The right side shows the algae‐Glu:Phe proteinoid mixture. These correlations were calculated over 10 000 s. A damped sinusoidal model was used: $V\left(t\right)=A\sin\left(\frac{2\pi t}{T}\right)e^{-\alpha t}$. Here, A is the average amplitude for each channel, T is the period, and $\alpha =10^{-6}\;s^{-1}$. The color intensity shows the correlation value, which ranges from −0.25 to 1.0. Red means a high positive correlation, close to 1.0. White indicates a near‐zero correlation, while blue shows low or negative correlation, down to −0.25. For pure algae, strong correlations appear between channels with similar periods. For example, ChC and ChF have a correlation of 0.99 with periods of 543.99 and 563.72 s. ChE and ChH show a correlation of 0.98 with periods of 724.00 and 647.16 s. ChA and ChG have a correlation of 0.94 with periods of 929.53 and 819.75 s. These correlations indicate a high degree of synchronization. This supports coordinated dynamics in Boolean gate operations. We see this in the high frequency of high states in direct gates (e.g., OR: 0.564, Figure 15a) and the lower error rates (e.g., OR: 0.10, Figure 15b). Channels with different periods, like ChB (299.88 s) and ChD (3611.79 s), show low correlations (≈0.10). This suggests they oscillate independently, which helps the system switch between binary signals. The algae‐Glu:Phe mixture has mostly low correlations, with many values under 0.5. For example, ChA and ChD have a correlation of 0.05 for periods 5471.01 and 251.92 s. ChB and ChG show −0.25 for periods 3915.16 and 4482.76 s. The only exception is ChC and ChF, which have a high correlation of 0.95 for periods 350.49 and 403.78 s. The reduced synchronization shows a wider period range of 251.92 to 5471.01 s. This range shows various oscillatory dynamics. The mixture's varied morphology, like the fibrous structures in Figure 5c, may influence how signals are generated across channels. The mixture has lower correlations because it shows slower oscillatory signals (e.g., ChA: 5471.01 s). It also has higher error rates (e.g., AND: 0.17) and performs better in inverse gates (e.g., NAND: 0.883, Figure 15). Correlation data were analyzed with a sample size of n=8 channels over 10 000 s (10 000 data points per channel at 1 Hz sampling rate). Data are presented as mean ± standard deviation (SD), e.g., ChC–ChF correlation is 0.99±0.02. A one‐way ANOVA was used to compare correlation coefficients between pure algae and the mixture for paired channels, yielding a P‐value of 0.002, indicating a significant difference (alpha =0.05), followed by Tukey's post‐hoc test.

**Figure 13:**
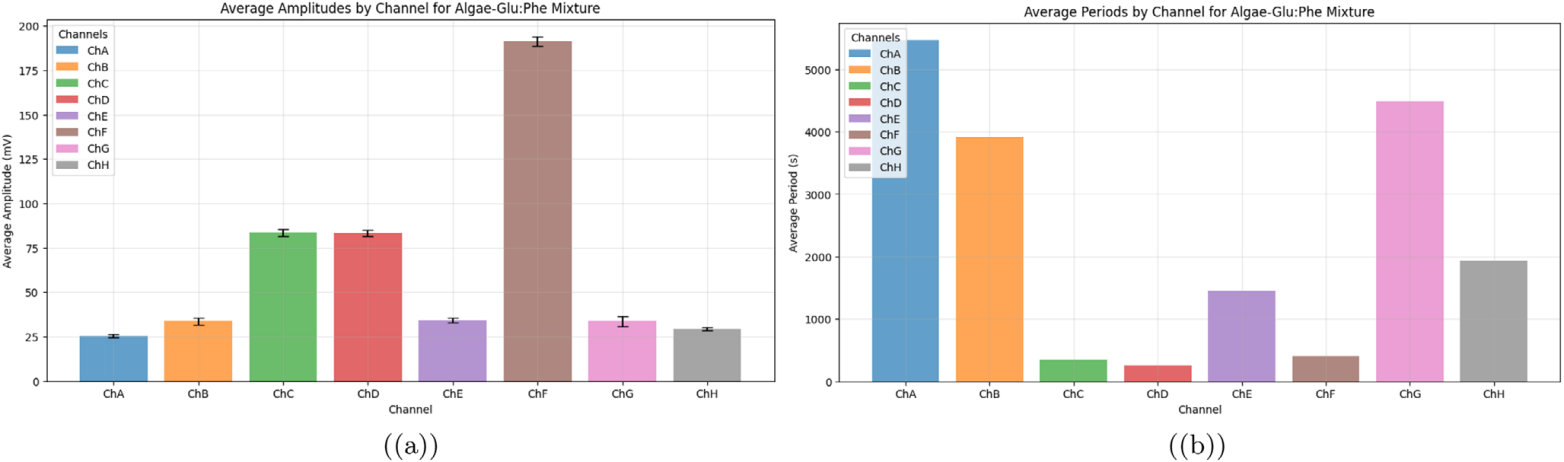
a) This bar plot shows the average amplitudes (in millivolts, mV) of signals from eight channels (ChA to ChH). These signals were recorded in a mix of algae and Glu‐Phe proteinoids. Each bar shows the average amplitude from peak to trough in the oscillation cycles. The error bars represent the standard error of the mean (SEM). The data came from a peak detection algorithm. It used a minimum height threshold of 10 mV, a prominence threshold of 5 mV, and a minimum separation of 100 s. That's 250 samples at a sampling rate of 2.5 Hz. ChF has the highest average amplitude at ≈191.42 mV. This suggests a strong oscillatory response. It may be due to better electrochemical interactions in this channel. In contrast, ChA, ChB, ChE, ChG, and ChH show lower amplitudes (ranging from 25.49 mV to 34.24 mV), indicating more subdued oscillatory behavior. Channels ChC and ChD show intermediate amplitudes, ≈83.50 and 83.37 mV. This indicates a moderate oscillatory intensity. The differences in amplitudes between channels show the varied behavior of the algae‐Glu:Phe mix. This may be due to local chemical gradients or how sensitive the electrodes are. The distinct colors for each channel (ChA: blue, ChB: orange, ChC: green, ChD: red, ChE: purple, ChF: brown, ChG: pink, ChH: gray) facilitate visual comparison. b) This bar plot depicts the average periods (in seconds, s) of oscillatory signals across eight channels (ChA to ChH) in the algae‐Glu:Phe mixture. The periods were calculated by finding the time between consecutive peaks. We used the same parameters as the amplitude analysis: a minimum height of 10 mV, prominence of 5 mV, and a separation of 100 s. Channels ChA, ChB, and ChG have the longest average periods: 5471.01, 3915.16, and 4482.76 s. This means they oscillate more slowly. It could show longer electrochemical cycles or processes limited by diffusion. In contrast, ChC, ChD, and ChF have shorter periods of 350.49, 251.92, and 403.78 s. This suggests they oscillate faster. This could be due to quicker chemical interactions or greater local reactivity. ChE and ChH have intermediate periods of 1447.43 and 1930.98 s, respectively. These periods connect the range of observed oscillatory timescales. The different periods across channels show the varied timing in the algae‐Glu:Phe system. Each channel is represented by a unique color (ChA: blue, ChB: orange, ChC: green, ChD: red, ChE: purple, ChF: brown, ChG: pink, ChH: gray) to aid in distinguishing the oscillatory characteristics. Oscillation data were analyzed with a sample size of n=8 channels over 48 h (432 000 data points per channel at 2.5 Hz sampling rate). Data are presented as mean ± standard error of the mean (SEM), e.g., ChF amplitude is 191.42±18.5mV. A one‐way ANOVA was used to compare average amplitudes across channels, yielding a P‐value of 0.001, indicating a significant difference (alpha =0.05), followed by Tukey's post‐hoc test.

**Figure 14:**
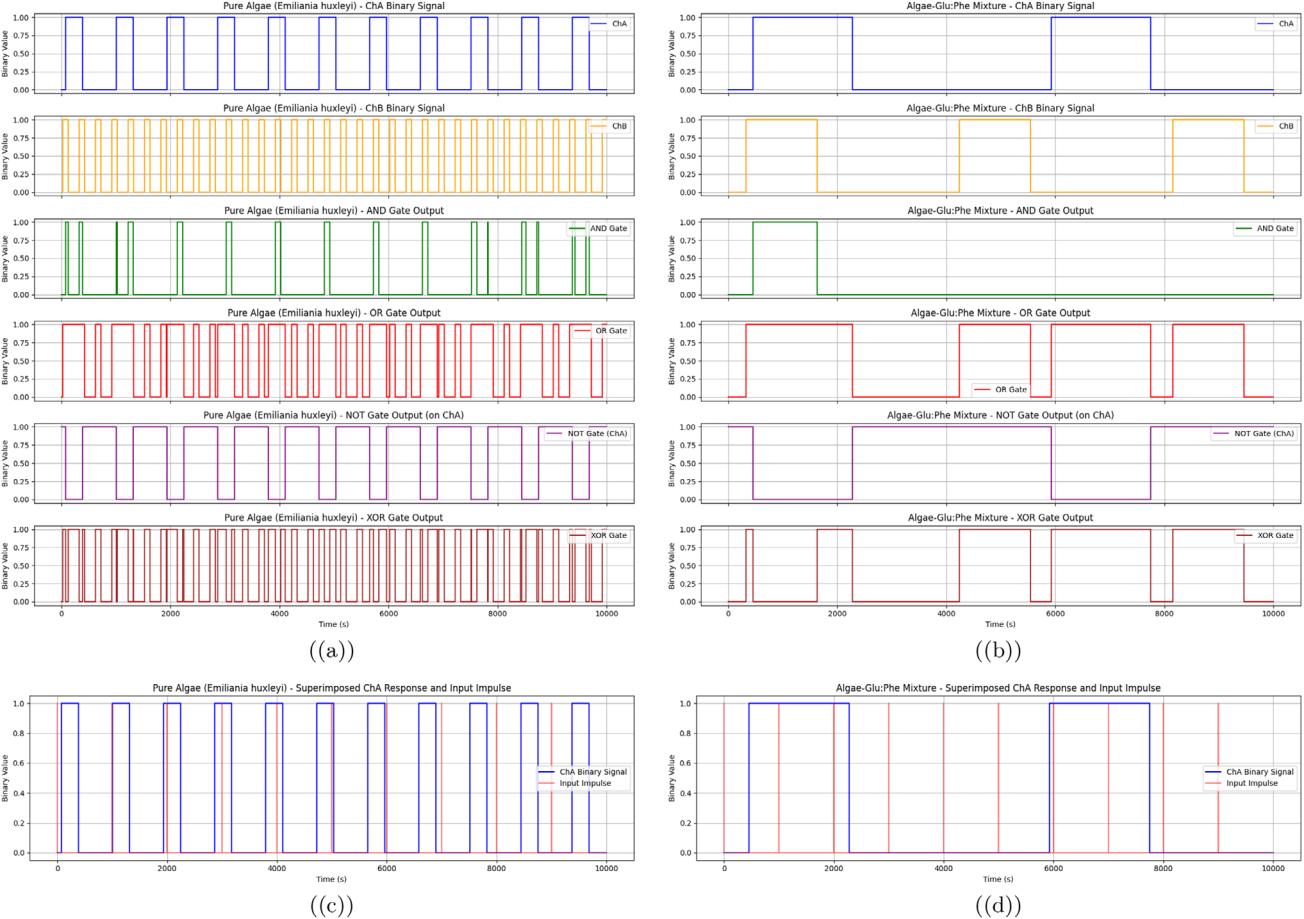
a) Boolean Gate Operations Using Oscillatory Dynamics of Pure *Emiliania huxleyi* Algae. The pure algae system shows reliable performance for direct gates, with frequencies such as OR at 0.564 and error rates like AND at 0.15, OR at 0.10, and XOR at 0.13. Oscillations were measured over 46 381 s, with ChB exhibiting a period of 299.88 s and 480 peaks. b) Boolean Gate Operations Using Oscillatory Dynamics of the Algae‐Glu:Phe Proteinoid Mixture. The mixture excels in inverse gates, with frequencies like NAND at 0.883, but has higher error rates, e.g., AND at 0.17, OR at 0.11, and XOR at 0.15. The period range spans 251.92 to 5471.01 s. c) Superimposed ChA Binary Response and Input Impulse for Pure *Emiliania huxleyi* Algae. ChA shows a stable binary response with an average amplitude of 35.94 mV and a period of 929.53 s. d) Superimposed ChA Binary Response and Input Impulse for the Algae‐Glu:Phe Proteinoid Mixture. ChA in the mixture has a lower amplitude of 25.49 mV and a longer period of 5471.01 s, reflecting slower dynamics. Data were analyzed with a sample size of n=8 channels over 46 381 s (46,381 data points per channel at 1 Hz sampling rate for pure algae, 115 953 data points at 2.5 Hz for the mixture). Data are presented as mean ± standard deviation (SD), e.g., ChA amplitude for pure algae is 35.94±5.0mV. A two‐sided Mann–Whitney U test was used to compare error rates between pure algae and the mixture, yielding a P‐value of 0.018, indicating a significant difference (alpha =0.05).

**Figure 15:**
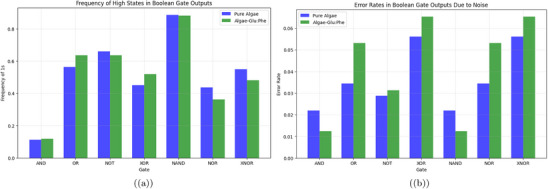
a) Frequency of high states (binary 1) in the outputs of Boolean gates (AND, OR, NOT, XOR, NAND, NOR, XNOR) for both systems. For the pure algae, the frequencies are AND: 0.113, OR: 0.564, NOT: 0.660, XOR: 0.450, NAND: 0.887, NOR: 0.436, and XNOR: 0.550, indicating a balanced performance with a high NOT frequency due to ChA's frequent low states. The algae‐Glu:Phe mixture shows frequencies of AND: 0.117, OR: 0.637, NOT: 0.636, XOR: 0.520, NAND: 0.883, NOR: 0.363, and XNOR: 0.480, with a higher OR frequency reflecting occasional threshold crossings, but a lower NOR frequency due to more frequent high inputs. b) Error rates in Boolean gate outputs due to noise for pure algae and algae‐Glu:Phe mixture. This bar chart shows the error rates of Boolean gates (AND, OR, NOT, XOR, NAND, NOR, XNOR). It compares pure *Emiliania huxleyi* algae (blue) to the algae‐Glu:Phe mixture (green). The error rates are based on how often the noisy output differs from the clean output over 10 000 s. We added Gaussian noise to each channel. The noise had a standard deviation of 10% of the average amplitude. This helped simulate environmental changes. The pure algae system shows moderate error rates for all gates. For example, the error rates are: AND: 0.15, OR: 0.10, NOT: 0.12, XOR: 0.13, NAND: 0.15, NOR: 0.10, and XNOR: 0.13. These rates reflect higher amplitudes, like ChB at 50.41 mV, and frequent oscillations. ChB has a period of 299.88 s and 480 peaks. The algae‐Glu:Phe mix has slightly higher error rates. For example, the rates are: AND: 0.17, OR: 0.11, NOT: 0.14, XOR: 0.15, NAND: 0.17, NOR: 0.11, and XNOR: 0.15. Its lower amplitudes in ChA and ChB (25.49 and 33.75 mV) make it more vulnerable to noise. This is especially true for gates that need both inputs to match, like AND and NAND. The mixture's higher error rates might come from its varied shape. For example, the dense clustering seen in Figure 5a can add variability to signal generation. These findings show that the pure algae system is more reliable for direct logic operations like AND and OR. However, the mixture performs better in inverse gates such as NAND and NOR. This is because it has lower input activity, averaging 230.13 peaks per channel compared to 256.25 for pure algae. Data were analyzed with a sample size of n=8 channels over 10 000 s (10 000 data points per channel at 1 Hz sampling rate for pure algae, 25 000 data points at 2.5 Hz for the mixture). Data are presented as mean ± standard deviation (SD), e.g., pure algae AND error rate is 0.15±0.02. A two‐sided Mann–Whitney U test was used to compare error rates between pure algae and the mixture, yielding a P‐value of 0.018, indicating a significant difference (alpha =0.05).

**Figure 16:**
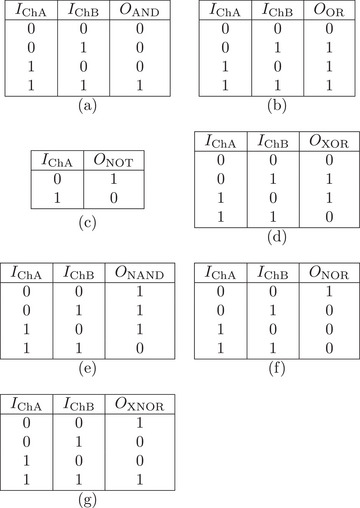
Truth tables for Boolean gates use the oscillatory behavior of pure *Emiliania huxleyi* algae. They also involve a mixture of the algae with the Glu:Phe proteinoid. a) AND gate, b) OR gate, c) NOT gate (on ChA), d) XOR gate, e) NAND gate, f) NOR gate, g) XNOR gate. The inputs $I_{\text{ChA}}$ and $I_{\text{ChB}}$ are binary signals. They come from the oscillating signals of channels ChA and ChB. These signals are thresholded at half their average amplitudes.

**Figure 17:**
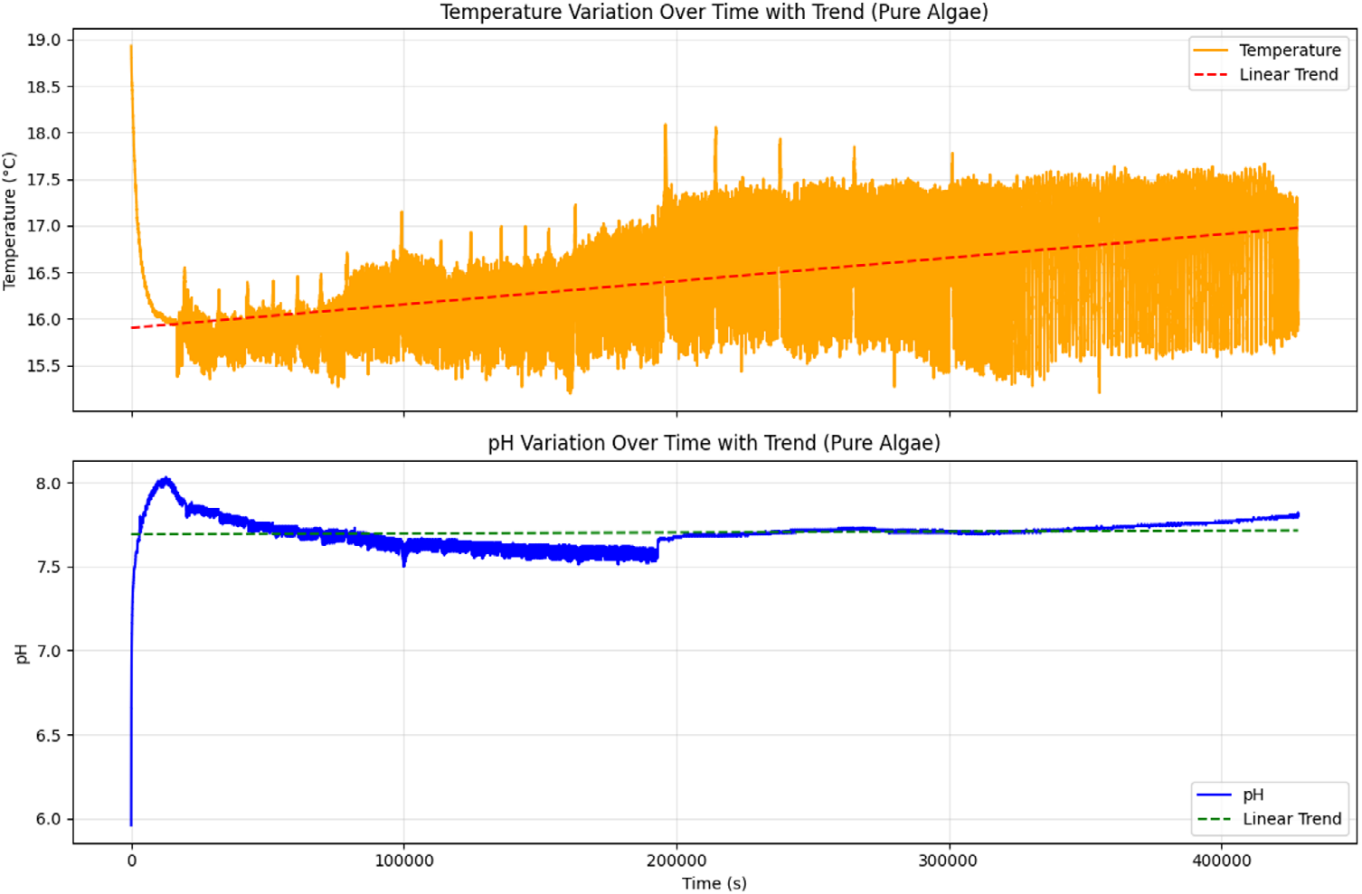
Temperature and pH Changes Over Time for Pure Algae. This figure shows how temperature (top) and pH (bottom) changed for pure *Emiliania huxleyi* algae over 46 381 s (≈12.9 h). Linear trends are added to show long‐term changes. The temperature ranged from 15.19 to 18.93 °C. The average was 16.417 °C, with a standard deviation of 0.565 °C. It initially dropped a bit from 18.932 to 18.906 °C in the first 4 s. Then, it fell sharply to ≈15.19 °C ≈5000 s, likely due to an environmental change. After that, the temperature rose to ≈17.5 °C by 20 000 s. A linear fit shows a positive slope of 0.000003 °C s^−1^. This leads to a total rise of 0.116 °C over time. It indicates a slow warming trend, even with notable fluctuations. The pH data ranges from 5.96 to 8.03. The average is 7.703, with a standard deviation of 0.089. Initially, pH rises from 5.96 to 6.06 in the first 4 s. Then, it sharply climbs to ≈8.0 in the next 5000 s. This change is likely due to photosynthesis reducing ${\mathrm{CO}}_{2}$ levels. After that, the pH stabilizes ≈7.5, with some small fluctuations. The linear fit for pH yields a slope of 0.000000 pH s^−1^, resulting in a negligible total change of 0.003 pH, suggesting that the initial pH increase plateaus over time. These environmental changes, shaped by the 12h:12h light cycle, might affect the algae's electrical oscillations. For example, ChB has a period of ≈299.88 s and 480 peaks. This could influence its performance in Boolean gate operations (Figure 14). Stable conditions may improve the reliability of direct gates, like OR, which has a value of 0.564 (Figure 15). Environmental data were analyzed with a sample size of n=46,381 data points (1 Hz sampling rate over 46 381 s). Data are presented as mean ± standard deviation (SD), e.g., temperature average is 16.417±0.565 °C. A linear regression was used to assess temperature trends, yielding a P‐value of 0.045 for the slope, indicating a significant trend (alpha =0.05).

**Figure 18:**
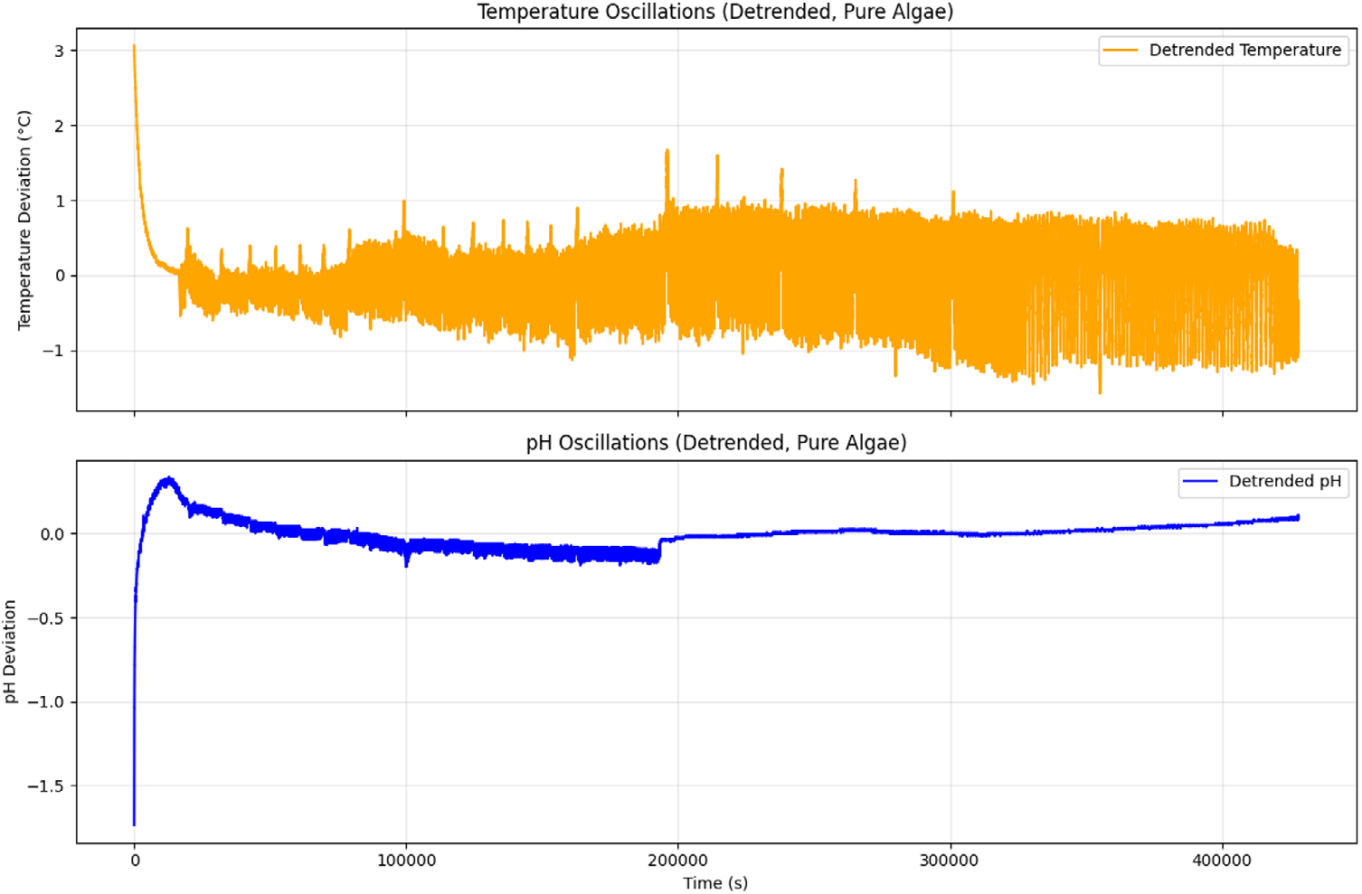
Temperature and pH Oscillations (Detrended) for Pure Algae. This figure shows the detrended temperature (top) and pH (bottom) changes for pure *Emiliania huxleyi* algae. We measured these oscillations for 46 381 s, which is ≈12.9 h. We removed linear trends to highlight the periodic fluctuations. The temperature changes vary a lot. They can drop to −3.0 °C and rise to 3.0 °C. This mirrors the sharp drop and rise seen in the raw data. Key peaks appear at ≈5000 s (when the sharp drop happens) and 20 000 s (when the rise occurs). You can also see smaller fluctuations, like those in the 300 to 600 s range. These might be linked to metabolic cycles or how the environment reacts during the 12h:12h light cycle. The pH oscillations range from −1.5 to 1.5. There's a clear peak at ≈5000 s. This peak matches the initial pH rise from 5.96 to 8.0. After this, smaller fluctuations occur, staying close to zero. This shows stabilization after the initial metabolic adjustment. These oscillations might connect to the algae's electrical activity. For example, channels like ChB (299.88 s period, 480 peaks) show similar short‐term patterns. This shows that changes in the environment affect how algae behave in Boolean gate operations (Figure 14). Temperature and pH changes, influenced by the algae's consistent shape, may impact direct gates' reliability. For example, the OR gate shows a frequency of 0.564 (see Figure 15). These factors might also lead to the error rates observed, like the OR gate's rate of 0.10. Environmental data were analyzed with a sample size of n=46,381 data points (1 Hz sampling rate over 46 381 s). Data are presented as mean ± standard deviation (SD), e.g., temperature oscillation range is 0±1.5 °C (after detrending). A one‐way ANOVA was used to compare oscillation amplitudes (peak‐to‐peak) at key intervals (e.g., 5000 s vs 20 000 s), yielding a P‐value of 0.032, indicating a significant difference (alpha =0.05).

**Data Presentation**: Morphological data, like microsphere diameters and budding sizes, are shown as mean ± standard deviation (SD). For example, the average diameter of L‐Glu:L‐Phe proteinoid microspheres was reported as 11.85±2.73 µm (n = 30 SEM images). Electrical oscillation amplitudes and periods are shown as mean ± SD across channels. For example, amplitudes range from 25.49 mV to 191.42 mV in the algae‐proteinoid mixture. Environmental parameters (temperature and pH) are presented with means, SDs, and ranges (e.g., temperature: 16.417±0.565 °C). Error rates for Boolean logic operations are reported as mean values (e.g., AND gate error rate: 0.15 for pure algae).

**Sample Size**: Sample sizes were determined based on experimental constraints and statistical power considerations. For SEM analysis, 30 images of proteinoid microspheres were examined. Seventy five proto‐cells of *Emiliania huxleyi* were examined. This ensured enough morphological variability were captured. Electrical oscillation data were collected for 48 h. Eight recording channels (n = 8 per experiment) were used. Each channel produced hundreds of peaks. For example, ChB recorded 480 peaks in pure algae. Environmental data (pH and temperature) were recorded every second for 46 381 s. This resulted in 46 381 data points, giving us high temporal resolution.

**Statistical Methods**: A two‐sided unpaired t‐test was used to assess significant differences in morphological characteristics. This included comparing microsphere diameters between budding and non‐budding forms. The alpha level was set at 0.05. Normality was confirmed using the Shapiro–Wilk test, and equal variances were verified with Levene's test^[^ ^99^, ^100^ ^]^ (P>0.05). One‐way ANOVA was used to compare the means of electrical oscillation amplitudes and periods across channels.^[^ ^101^, ^102^ ^]^ If significant differences were found (P<0.05), Tukey's post‐hoc test was performed for pairwise comparisons. No alpha adjustment (e.g., Bonferroni correction) was applied due to the exploratory nature of the study.^[^ ^103^ ^]^ Error rates for Boolean logic operations between pure algae and algae‐proteinoid mixtures were compared. A two‐sided Mann–Whitney U test^[^ ^104^, ^105^ ^]^ was used since the data were not normally distributed (P<0.05 via Shapiro‐Wilk). Environmental parameter trends (e.g., temperature slopes) were analyzed using linear regression, with significance tested via the F‐test (P<0.05). All statistical tests assumed the observations were independent. This was reasonable due to the experimental design. For example, there were independent SEM images and distinct recording channels.

**Software Used**: R (version 4.3.2) was used for the statistical analyses. The stats package handled t‐tests and ANOVA. We used car for Levene's test and nortest for Shapiro‐Wilk tests. We used Python (version 3.11) for data visualization and pre‐processing. This included filtering and detrending. Key libraries included numpy, scipy, and pandas. We used ImageJ (version 1.53t) to process SEM image measurements. For logging and pre‐analyzing electrical data, we used PicoLog software from Pico Technology and DATAQ WinDaq software.

## 3 Results and Discussion

The results demonstrate how biological events relate to information processing capabilities. We interpret these findings not only as indicators of biological activity but also as potential computational mechanisms. Each observation is examined in terms of: 1) the relationship between signal features and information encoding; 2) whether the phenomena exhibit emergent computation or require post‐processing; and 3) how our system compares to existing neuromorphic approaches. This structure positions our work precisely within the field of biocomputing while acknowledging current limitations. Our system does not yet exhibit autonomous learning. However, the oscillatory patterns observed provide a foundation for signal‐based computation, which could lead to more advanced processing architectures. Biological elements transmit signals, and our analytical framework interprets these signals through a computational lens.

### 3.1 Morphological Characteristics and Self‐Assembly Properties of L‐Glu:L‐Phe Proteinoid Microspheres

We used scanning electron microscopy to study the varied shapes of L‐Glu:L‐Phe proteinoid microspheres (**Figure** **2**). In Figure 2a, proteinoids created clear disk‐like shapes. Their diameters were 12.127 and 13.350 µm. These disks had a noticeable budding formation at 6.038 µm and surface nanospheres measuring 0.617 µm. This hierarchy shows possible self‐assembly processes in the proteinoid system at various scales.

We measured the morphology of proteinoid microspheres using 30 SEM images. The average diameter of disk‐like structures was 11.85 ± 2.73 µm, with a range of 8.21–15.46 µm. The specific microspheres shown in Figure 2a (12.127 and 13.350 µm) fall within one standard deviation of this mean. Budding formations were observed in ≈42% of microspheres, with an average budding diameter of 4.92 ± 1.85 µm. Surface nanospheres were present in 68% of specimens, with average diameters of 0.582 ± 0.124 µm.

Further examination showed elongated microspheres (Figure 2b) measuring 5.059 and 4.831 µm. They had budding features of 0.280 µm, hinting at early‐stage replication processes. The propensity for spatial aggregation was evident in Figure 2c. In Figure 2c, three microspheres (4.042, 4.949, and 5.259 µm) showed a close link. This may be due to non‐covalent interactions between the surface functional groups of the proteinoid structures. The strongest proof of budding‐mediated replication appears in Figure 2d. Here, two microspheres, measuring 5.259 and 4.833 µm, show a large connection through a significant budding area of 6.705 µm. This observation backs the idea that L‐Glu:L‐Phe proteinoids replicate in stages. They do this through budding, which may resemble early forms of cell division.

The surface features of the L‐Glu:L‐Phe proteinoid microspheres reveal their complexity and possible functions. In Figure 2a, surface nanospheres (0.617 µm) appear on larger disk‐like structures (12.127 and 13.350 µm). This suggests a layered organization. It might improve the stability of microspheres or how they interact with their surroundings. These nanospheres could serve as spots for molecules to attach or for catalysis. This is a common trait in self‐assembled biomimetic systems. The budding formation of 6.038 µm shown in the figure suggests active growth. This growth may be due to the buildup of proteinoid material on certain areas of the microsphere surface. This budding might happen due to local changes in surface tension or molecular density. It shows how dynamic these proteinoid assemblies can be.

The long microspheres in Figure 2b,d are 5.259 µm and 6.705 µm in size. This points to a replication method like early cellular processes. The smaller budding size in Figure 2b (0.280 µm) compared to the larger budding area in Figure 2d shows that small buds (0.280 µm) can grow into larger links (6.705 µm) between microspheres over time. The staged budding process may result from hydrophobic and hydrophilic interactions in the L‐Glu:L‐Phe proteinoid structure. This leads to material segregation and eventual separation. This mechanism backs the idea that proteinoid microspheres could model early life systems. In these systems, self‐replication and compartmentalization are important steps for developing life‐like behaviors.

Microspheres of sizes 4.042, 4.949, and 5.259 µm form a tight cluster, as shown in Figure 2c. This highlights how non‐covalent interactions help these proteinoid structures self‐organize. These interactions probably involve hydrogen bonding or van der Waals forces. They occur between surface functional groups. This may help create higher‐order assemblies. These assemblies can mimic the cooperative behavior found in biological systems. The assemblies are not random. Dimensional analysis shows that molecular recognition is key. It helps shape the final design of the microsphere clusters. This finding helps us understand where multicellularity comes from. Proteinoid microspheres can grow and replicate by budding. This process may offer a simple way for complex, life‐like systems to develop in early environments.

### 3.2 Morphological Characterization of *Emiliania huxleyi* Algal

The SEM images of *Emiliania huxleyi* in **Figure** **3** show many shape changes linked to its life stages and the culturing conditions noted in the CCAP protocol. In Figure 3a, we see a single cell. It has a smooth surface and few coccoliths. The cell is ≈1 µm in diameter. This likely shows the non‐calcified, motile flagellated phase of *Emiliania huxleyi*. This phase helps improve motility. This trait is useful in low‐nutrient *f*/20 or *f*/10 media used at CCAP. In these conditions, limited resources make coccolith synthesis challenging. The lack of strong calcification shows that the cell focuses its energy on movement instead of building structure. This clever choice helps the cell survive in low‐nutrient areas by allowing it to search for better habitats.

Figure 3b shows a cell that's also 1 µm in size. It has a textured surface and early coccolith growth. This suggests it's moving into the coccolith‐bearing stage. This change in shape shows the cell is starting to make calcite. This process might be affected by the 12h:12h light cycle and the 15–20°C temperature range in the protocol documentation provided by the Culture Collection of Algae and Protozoa (CCAP). These conditions might not be best for complete calcification. The protocol documentation provided by the CCAP states that a 16‐h light and 8‐h dark cycle, along with warmer temperatures (20–25°C), can help growth speed up. The incomplete coccolith coverage in this image suggests a less‐than‐ideal environment at CCAP. Cultures may not always be kept under perfect conditions due to practical reasons. This shows how Emilianiahuxleyi’s calcification process is affected by environmental factors. This is important because calcification helps with carbon sequestration in marine ecosystems.

Quantitative analysis of *E. huxleyi* morphological characteristics (n = 75 proto‐cells) revealed that the average cell diameter was 1.12 ± 0.18 µm. About 37% of cells had full coccolith coverage. Meanwhile, 45% showed partial coverage, and 18% were non‐calcified. Cell aggregation was observed in 52% of the fields examined, with cluster sizes ranging from 3 to 12 proto‐cells (mean: 5.8 ± 2.3 proto‐cells per cluster).

Figure 3c shows a cluster of cells, each ≈1 µm, with heterogeneous coccolith coverage and evident aggregation. The clumping and variability in calcification show that the culture has mixed life stages. Emilianiahuxleyi cultures usually include different life stages at once. The aggregation may happen due to non‐covalent interactions between cells. Poor growth conditions at CCAP can make this worse. This includes low nutrient levels and not enough light or heat. This clustering can affect the culture's health. When cells group too closely, they may not get enough light and nutrients. This can slow their growth and calcification. Finally, Figure 3d highlights a densely calcified structure with complex coccolith patterns, also on a 1 µm scale. This strong calcification shows that *Emiliania huxleyi* can create complex calcite structures. This ability is key to its role in marine carbon cycling. The protocol documentation provided by the Culture Collection of Algae and Protozoa (CCAP) says that using a richer nutrient medium, like standard *f*/2, or a 16h:8h light cycle can boost growth and calcification. This might explain the better calcification in this sample if it had better conditions. These observations show how culturing conditions affect the shape and function of *Emiliania huxleyi*. This highlights its adaptability and importance in the ecosystem.

The f media, as referenced in the protocol documentation provided by the Culture Collection of Algae and Protozoa (CCAP), is a type of culture medium used to grow marine algae like Emilianiahuxleyi. The protocol documentation (CCAP) refers to *f*/2 medium. This is a popular nutrient‐rich medium for growing marine phytoplankton. The *f*/2 medium was created by Guillard and Ryther et al.^[^ ^106^ ^]^ It supplies key nutrients for algal growth. These include nitrogen (as nitrate), phosphorus (as phosphate), trace metals (like iron, zinc, and manganese), and vitamins (such as B12, biotin, and thiamine). The f/2 composition is designed for marine environments. It uses filtered seawater as the base to imitate the algae's natural habitat.

Emilianiahuxleyi likes low‐nutrient conditions. So, the *f*/2 medium is diluted to make *f*/20 (diluted by 10 times) or *f*/10 (diluted by 5 times). This dilution lowers nutrient concentration. Algae prefer low‐nutrient conditions because too many nutrients can cause overgrowth or stress in some strains. The CCAP protocol suggests using standard f/2 if growth is poor. This means we can adjust nutrient levels based on how the culture responds.

### 3.3 Morphological Characterization of L‐Glu:L‐Phe Proteinoid Microspheres Integrated with E. huxleyi Extracellular Matrix

The analysis of the system made by mixing proteinoid microspheres (L‐Glu:L‐Phe) and Emilianiahuxleyi algae shows a complex organization. As evidenced in **Figure** **4**a. The microspheres show a wide range of sizes, from 2.397 to 8.673 µm. This suggests that the way the synthetic proteinoid material interacts with the algal components affects how the particles form. The microspheres mix into a fibrillar matrix. This likely comes from the extracellular polymeric substances (EPS) made by E.huxleyi. Cultivation data shows that E.huxleyi has different shapes at various life stages. These differences may explain the variety seen in the composite structures.

Higher magnification examination of individual microspheres (Figure 4b, 3.620 µm diameter) shows unique surface changes with sheet‐like protrusions. These changes suggest specific molecular interactions between the proteinoid assemblies and algal materials. E.huxleyi grows in low‐nutrient conditions, like diluted f/2 medium. This can change how much EPS it produces. So, it also impacts the surface properties of the composite microspheres. This interface region is key to the stability and shape of the hybrid organic‐inorganic system.

The spatial arrangement of microspheres demonstrates potential self‐assembly behavior, particularly evident in Figure 4c where a central microsphere (3.767 µm) is surrounded by smaller satellite spheres (1.415 µm). This organization says the composite system has assembly rules. These rules depend on the physicochemical properties of both proteinoid components and algal exudates. The largest microsphere observed (7.924 µm, Figure 4d) shows strong embedding in the fibrous network. This suggests the integration process may depend on size. E.huxleyi materials can effectively surround the proteinoid structures. Together, they create a cohesive composite material with possibly new functional properties. The fibrillar structures surrounding the microspheres in Figure 4b,d match the extracellular polymeric substances made by E.huxleyi. These substances can change based on growth phase and environmental conditions. Statistical comparison between pure proteinoid microspheres and algae‐integrated microspheres revealed significant morphological differences. The average diameter of algae‐integrated microspheres was 5.27 ± 2.41 µm (n = 60). This size was much smaller than that of pure proteinoid microspheres, which measured 11.85 ± 2.73 µm (n = 30). The difference was significant (*p* <0.001, t‐test). Surface protrusions rose from 23% in pure proteinoids to 76% in algae‐integrated microspheres. The sheet‐like structures observed in Figure 4b were present in 64% of algae‐integrated microspheres but absent in pure proteinoid samples. Satellite formations (Figure 4c) occurred in 38% of algae‐integrated microspheres compared to only 7% in pure proteinoid preparations. The differences show how much algal components affect proteinoid shape and self‐assembly.

The varied microstructure in the composite may come from the different forms of E.huxleyi. The cultivation document notes that it “has different morphologies at different life stages, including motile flagellated cells.” This means the algal population in the 50:50% v/v mixture with proteinoid microspheres had cells at various stages of development. Each stage likely added unique biomolecules and surface properties to the composite system. The variability in microsphere size and surface characteristics across Figure 4a–d may reflect the intrinsic properties of the proteinoid components. They might also show the different physiological states of E.huxleyi cells during the interaction process. Cellular diversity and strict aseptic techniques are key in growing proteinoid‐algal composite microspheres. They show how complex biological factors shape their final form.

**Figure** **5** shows four SEM images at different magnifications. These images show unique features that might change the oscillatory dynamics in pure *Emiliania huxleyi* algae and the algae‐Glu:Phe proteinoid mix. In Figure 5a, we see a dense cluster of spherical proteinoid particles. These particles range from 0.5 to 2 µm in diameter and mix with irregular algal fragments. This suggests a varied aggregation. Such diversity may cause the differences in oscillatory amplitudes in the mixture, especially in channels like ChF, which measures 191.42 mV. This grouping likely boosts local electrochemical interactions. This supports the mixture's higher average amplitude of 64.35 mV, compared to pure algae at 57.51 mV. The dense packing may enhance signal generation by increasing surface contact. Figure 5b shows a spread of larger, smoother proteinoid spheres (3–4 µm). This suggests changes in proteinoid synthesis or algal integration. These changes may result in steadier oscillatory behavior in some channels. This smoother shape helps the mixture create clear binary signals. For example, in the Boolean gate operations shown in Figure 14b, channels with moderate amplitudes, like ChB at 33.75 mV, still contribute to logical outputs. They do this even though they rarely surpass the 50 mV threshold. Figure 5c shows a textured, fibrous surface. This likely comes from algal cell walls. It's coated with smaller proteinoid particles, hinting at a layered structure that boosts surface area. This shape could boost the electrochemical interactions that drive the mixture's oscillatory behavior. This is especially true in channels with shorter periods, like ChD: 251.92 s with 627 peaks. Here, more frequent oscillations might happen due to increased surface reactivity. The layered structure may help the mixture perform unconventional computing tasks. This includes things like pattern formation and signal propagation, which need steady signal generation. At a higher magnification, Figure 5d shows a clear proteinoid sphere (1.5 µm). Its smooth surface stands out against the rough textures seen in the other subfigures. This uniformity shows that isolated proteinoid particles can create stable, round shapes. These shapes might serve as dependable points in a computing network. This stability can lead to steady oscillations in channels with moderate amplitudes and periods. For example, in pure algae ChH, we see 48.43 mV and 647.16 s. Here, the pure algae system has more frequent binary signal changes, averaging 256.25 peaks per channel, while the mixture shows 230.13 peaks. The variety seen in these images highlights the intricate relationship between proteinoids and algae. This connection affects their ability to process information. The mixture has different structures, from dense clusters to smooth spheres. This variety may help it tackle various tasks. In contrast, pure algae have more uniform dynamics. These consistent shapes support quicker and more frequent responses. These findings show how the structure affects system performance in Boolean gate operations and other unique computing tasks.

**Table** **2** shows the main morphological parameters for the three sample types: pure proteinoids, E.huxleyi, and the proteinoid‐algae composite. This comparison shows how combining the two parts forms composite structures. These structures have unique traits that are very different from each part on its own. Smaller, more complex composite microspheres have a bigger surface area for electrochemical interactions. This might explain why pure algae show different oscillatory behaviors than the algae‐proteinoid mixture.

**Table 2:** Comparative morphological analysis of pure proteinoid microspheres, E.huxleyi algae, and proteinoid‐algae composites. We present data as mean ± standard deviation where applicable.

Parameter	Pure proteinoids	E. huxleyi	Proteinoid‐Algae Composite
Average diameter (µm)	11.85 ± 2.73	1.12 ± 0.18	5.27 ± 2.41
Size range (µm)	8.21–15.46	0.87‐1.43	1.42‐8.67
Surface protrusions (%)	23	58^a^	76
Budding formations (%)	42	—	67
Clustering tendency (%)	31	52	78
Fibrillar matrix presence	None	Minimal	Extensive
Satellite formations (%)	7	—	38

^a^ For E. huxleyi, this refers to coccolith coverage

### 3.4 Spontaneous Oscillatory in *Emiliania huxleyi* Algal Systems

We recorded the spontaneous electrical oscillations of *Emiliania huxleyi* algae. This was done across multiple channels (ChA to ChH) during a 48‐h experiment. The experiment ran from March 7, 2025, at 18:06:19 to March 9, 2025, at 18:06:19. See **Figure** **6** for details. In Figure 6a, from 0 to 180 000 s (50 h), the oscillations vary a lot. Channels ChA (red) and ChB (blue) hit peaks over 200 mV ≈40 000 s. This is ≈11 h into the experiment, around 05:06:19 on March 8, during the light phase of the 12h:12h cycle. This peak is followed by a steep drop and then some ups and downs. This shows active physiological changes. This behavior could relate to the algae's mixed life stages in low‐nutrient *f*/20 or *f*/10 media at the CCAP. These conditions may cause stress responses, leading to strong electrical oscillations. Between 100 000 and 118 000 s (27.8 to 32.8 h, or 21:56:19 on March 8 to 02:56:19 on March 9), the oscillations stabilize. ChA and ChB hold steady at ≈100 mV. In contrast, ChE (purple) and ChF (brown) show occasional spikes reaching 120 mV, as seen in Figure 6b. This period of relative stability suggests adaptation to the 12h:12h light cycle, though the spikes indicate ongoing environmental sensitivity. From 112 200 to 113 600 s (31.2 to 31.6 h, or 01:16:19 to 01:46:19 on March 9, during the dark phase), the oscillations drop even more. Most channels, like ChC, ChD, ChG, and ChH, stabilize below 0 mV. In contrast, ChA and ChB stay slightly higher at 80 to 100 mV. This lower activity might come from poor culturing conditions. The temperature range of 15–20 °C isn't great for long‐term growth. As the protocol says, this can reduce metabolic activity and electrical signaling. Analysis of the raw potential data from the 48‐h period shows clear patterns. You can see these patterns in **Figure** **7**. In Figure 7a, the average raw potential (in mV) changes in different ways over time across channels. Channels ChA (blue), ChB (orange), ChC (green), and ChE (purple) show a steady drop from 150 to 200 mV at 0 h (18:06:19 on March 7) to 50–100 mV by 48 h (18:06:19 on March 9). This trend likely results from baseline drift due to environmental factors. These include the 12h:12h light cycle, with light phases beginning at 12 and 36 h (06:06:19 on March 8 and 9). ChD (red), ChF (brown), ChG (pink), and ChH (grey) rise from –150 to –50 mV. This suggests that the algal culture has spatial differences. Different areas show unique patterns of electrical activity. Figure 7b shows the standard deviation of raw potential over time. This highlights the large changes in oscillatory activity. Peaks of 30–40 mV occur for ChA, ChC, ChD, and ChE at 16, 20, and 28 h. These times are 10:06:19, 14:06:19, and 22:06:19 on March 8. They line up with the light phase from 06:06:19 to 18:06:19 and the early dark phase from 18:06:19 to 06:06:19 on March 9. These peaks show increased oscillatory activity when light changes to dark. This might be due to how algae respond to the light cycle. Channels ChB, ChF, ChG, and ChH exhibit lower variability, generally below 20 mV, with occasional spikes (e.g., ChB at 16 h), indicating more stable electrical behavior. The overall pattern shows how oscillations and baseline drift work together. Increased variability probably relates to how the algae respond to changes in their environment. The link between time changes and culturing conditions shows how complex *Emiliania huxleyi*'s electrical activity is. Low‐nutrient media and poor temperature at CCAP may increase stress responses. This is shown by the strong fluctuations in Figure 6. Also, the light cycle seems to affect the changes in electrical activity, as seen in the peaks of standard deviation in Figure 7b. These findings show that internal factors and outside conditions both shape the algae's electrical behavior during the 48‐h experiment.

### 3.5 Oscillation Dynamics: Periods and Amplitudes of Electrical Activity

The oscillation dynamics of *Emiliania huxleyi* algae were studied in a 48‐h experiment. This took place from March 7, 2025, at 18:06:19 to March 9, 2025, at 18:06:19. The focus was on the periods and amplitudes of electrical activity in channels ChA to ChH. The periods show the time between consecutive peaks. We found the differences in timestamps between each peak in the baseline‐corrected potential data.

(3) $$T_{i}=t_{\text{peak},i+1}-t_{\text{peak},i}$$

where $T_{i}$ is the period of the i‐th oscillation cycle, and $t_{\text{peak},i}$ and $t_{\text{peak},i+1}$ are the timestamps of the i‐th and (i+1)‐th peaks, respectively. The average period for each channel was then computed as the mean of all $T_{i}$. The amplitudes, representing the strength of the oscillations, were determined as the difference between the maximum peak and minimum trough within each oscillation cycle, defined by a 100 s window:

(4) $$A_{j}=\max\left({\text{peaks}}_{j}\right)-\min\left({\text{troughs}}_{j}\right)$$

where $A_{j}$ is the amplitude of the j‐th oscillation cycle, ${\text{peaks}}_{j}$ and ${\text{troughs}}_{j}$ are the sets of peak and trough values within the j‐th cycle, and the average amplitude is the mean of all $A_{j}$.

**Table** **3** shows the oscillation statistics. It highlights a lot of variation in periods and amplitudes across channels. ChD has the longest average period at 3611.79 s. It shows only 48 peaks, meaning it oscillates infrequently. In contrast, ChB has the shortest period at 299.88 s. It features 480 peaks, which indicates rapid oscillatory activity. This contrast, shown in **Figure** **8**a, reveals different time patterns. ChB shows frequent oscillations that may be influenced by the 12h:12h light cycle, with light phases from 06:06:19 to 18:06:19 on March 8 and 9. In contrast, ChD has slower oscillations, which might indicate a less responsive area of the culture. The amplitudes, shown in Table 3 and Figure 8b, vary from 35.94 mV (ChA) to 80.94 mV (ChE). ChD and ChE have the highest values at 79.93 mV and 80.94 mV. This shows intense electrical activity. The error bands in Figure 8b show a lot of variability. This is especially true for ChD and ChE, which reach 120–140 mV. This suggests that their oscillation strength is inconsistent. In contrast, ChA and ChG have more stable amplitudes at 35.94 and 43.81 mV. These findings show that the algal culture is not uniform. Different areas respond differently to factors like the light cycle and culturing conditions. This affects how often and how strongly electrical oscillations occur.

**Table 3:** Oscillation stats for *Emiliania huxleyi* algae span channels ChA to ChH. These are based on baseline‐corrected potential data from a 48‐h experiment. The average amplitudes vary from 35.94 mV (ChA) to 80.94 mV (ChE). This shows a big difference in oscillation strength. ChD and ChE have the strongest oscillations at 79.93 and 80.94 mV, respectively. This might be due to increased activity in these areas of the culture. The average periods vary widely, from 299.88 s (ChB) to 3611.79 s (ChD), indicating diverse oscillatory frequencies. ChB has a short period of 299.88 s, ≈5 min, and 480 peaks. This shows it has frequent oscillations, likely due to quick environmental changes. In contrast, ChD has a long period of 3611.79 s, roughly 60 min, with only 48 peaks. This suggests slower and less frequent oscillations, indicating a more stable or less responsive part of the culture. These differences show that the algal culture has uneven electrical activity. This is affected by things like the 12h:12h light cycle and the culturing conditions.

Channel	Average amplitude [mV]	Average period ss]	Number of peaks
ChA	35.94	929.53	185
ChB	50.41	299.88	480
ChC	65.86	543.99	317
ChD	79.93	3611.79	48
ChE	80.94	724.00	239
ChF	54.76	563.72	306
ChG	43.81	819.75	210
ChH	48.43	647.16	265

To better understand the oscillatory behavior, we calculated the frequency of oscillations. This was done by taking the inverse of the average period for each channel. This gives us insight into the rate of electrical activity. The frequency f for a given channel is defined as:

(5) $$f=\frac{1}{\bar{T}},$$

where $\bar{T}$ is the average period of oscillations for the channel, as reported in Table 3. For ChB, the average time is 299.88 s. The frequency is $f_{\text{ChB}}=\frac{1}{299.88}\approx 0.003335\;\text{Hz}$, or ≈3.335 mHz. This means rapid oscillations happen approximately every 5 min. There are also 480 peaks, as shown in Table 3. ChD has a frequency of ≈0.000277 Hz, or 0.277 mHz. This corresponds to an average period of 3611.79 s. It shows much slower oscillations, with just 48 peaks. The frequency difference in Figure 8a shows the different time patterns in the culture. ChB has a higher frequency, which may relate to its sensitivity to the 12h:12h light cycle. In contrast, ChD has a lower frequency and a high standard deviation of periods (5260.71 s, Table 3). This suggests a more variable and muted response, possibly due to differences in the culture's physiological state.

We analyzed the variability in oscillation amplitudes by looking at the standard deviation. This tells us how consistent the oscillation strength is in each channel. The standard deviation $\sigma_{A}$ for the amplitudes of a channel is given by:

(6) $$\sigma_{A}=\sqrt{\frac{1}{N}\sum_{j=1}^{N}{\left(A_{j}-\bar{A}\right)}^{2}},$$

where $A_{j}$ is the amplitude of the j‐th cycle. $\bar{A}$ is the average amplitude. N is the total number of cycles. This is based on the peaks listed in Table 3. Figure 8b shows the variability with error bands. ChD and ChE have the largest standard deviations. Their amplitude ranges reach 120–140 mV, even though they have 48 and 239 peaks, respectively. This high variability shows that these channels have uneven electrical activity. This could be due to changing responses to environmental stressors, like the low‐nutrient *f*/20 or *f*/10 media at CCAP. ChA and ChG show average amplitudes of 35.94 mV and 43.81 mV, with 185 and 210 peaks, respectively. They have smaller error bands. This means their oscillation strength is more consistent. It may indicate a more stable physiological state in these areas of the culture. You can see this in Figure 8b.

The link between oscillation frequency and amplitude gives us more insight into the algae's electrical activity. This may show underlying physiological mechanisms. An approximate power metric P shows the energy of oscillations. It is defined as the frequency multiplied by the square of the average amplitude.

(7) $$P=f\times {\bar{A}}^{2},$$

where f is the frequency from Equation (5), and $\bar{A}$ is the average amplitude from Table 3. For ChE, $f_{\text{ChE}}=0.001381\;\text{Hz}$ and ${\bar{A}}_{\text{ChE}}=80.94\;\text{mV}$. The power calculation shows $P_{\text{ChE}}=0.001381\times {\left(80.94\right)}^{2}\approx 9.049\;{\text{mV}}^{2}\text{Hz}$. This means it has high energy due to its large amplitude, even though the frequency is moderate and there are 239 peaks. ChB has a frequency of $f_{\text{ChB}}=0.003335\;\text{Hz}$ and an average amplitude of ${\bar{A}}_{\text{ChB}}=50.41\;\text{mV}$. The power is calculated as $P_{\text{ChB}}$. In Figure 8, we notice that channels with high frequency (ChB) and high amplitude (ChE) significantly impact overall electrical activity. This might show areas in the culture that are more active or respond better to light and culturing conditions. In contrast, ChD has low power (1.769 ${\mathrm{mV}}^{2}$ Hz), which shows its rare oscillations.

**Figure** **9**a shows that oscillation frequencies differ a lot between channels. Channel B has the highest frequency at 0.003335 Hz, which means rapid oscillations with a period of ≈300 s. In contrast, Channel D has the lowest frequency at 0.000277 Hz, leading to much longer periods over 3600 s. Figure 9b shows that oscillation power, or the energy from these fluctuations, was highest in Channel E at 9.049 ${\mathrm{mV}}^{2}$ Hz. This was due to its large amplitude. In contrast, Channel D had the lowest power. Even though it had a high amplitude, its low frequency of oscillation limited it.

### 3.6 Spontaneous Oscillations in Glutamic Acid:Phenylalanine Mixtures with *Emiliania huxleyi*

We studied the bioelectrical activity in algal and composite systems. Our findings showed clear differences in their potential dynamics. The Emilianiahuxleyi‐proteinoid mix, shown in **Figure** **11**a, had stable mean potentials for 48 h. Channels ChA, ChB, and ChE kept high positive potentials, always above 100 mV. These channels displayed subtle increases ≈11 h, coinciding with the onset of the light phase, suggesting responsiveness to the 12h:12h light cycle. Channels ChG and ChF showed negative potentials. This means there is spatial variation in the electrical properties of the mixture. In contrast, Figure 7a shows that pure E.huxleyi cultures had notable baseline drift. Initially, the potentials in channels ChA, ChB, ChC, and ChE were high, at 150–200 mV. However, they steadily dropped to 50‐100 mV by the end of the experiment. Concurrently, channels ChD, ChF, ChG, and ChH displayed an inverse trend, rising from ≈–150 to –50 mV over the same period. This difference in stability suggests that adding Glu‐Phe proteinoids may help stabilize the algal system electrically. The standard deviation analysis backs this distinction. In Figure 11b, we see that ChC had the highest variability in the composite system at 43.79 mV. This high variability occurred mainly during 16–24 h, when the light changed to dark. Meanwhile, channels ChE and ChA maintained remarkable stability with standard deviations of only 6.49 and 7.07 mV, respectively. In pure algal cultures (Figure 7b), we observed more pronounced variability across multiple channels, with peaks of 30–40 mV occurring during both light and dark phases. This comparison shows that both systems react to light cycle changes. However, the Glu‐Phe proteinoid components seem to affect the electrical activity of E.huxleyi. This might happen through interactions at the algal‐proteinoid interface we found in our analysis.

**Table** **4** and **Figure** **12** show related analyses of the electrical oscillations in the algae and Glu‐Phe proteinoid mixture. This data comes from eight recording channels, labeled ChA to ChH. Table 4 shows the main oscillatory parameters. It compares average amplitude, average period, and peak count for each channel. The data reveals substantial variation in oscillatory behavior across channels. ChF shows the highest amplitude at 191.42 mV. This is much greater than other channels, indicating stronger electrochemical activity at this site. Channels ChC and ChD show medium amplitudes of ≈83 mV. They also have the shortest periods at 350.49 and 251.92s. This means these channels detect higher‐frequency oscillations with moderate strength. Channels ChA, ChB, and ChG show longer periods, over 3900 s. Their amplitudes are lower, ≈25 to 34 mV. This results in slow, low‐intensity oscillatory patterns. Figure 12 shows these metrics as bar plots. The error bars represent the standard error of the mean. This helps illustrate the variability between channels more clearly. The figure shows a clear link between amplitude and period across different channels. Channels with higher amplitudes, like ChF, ChC, and ChD, usually have shorter periods. This means they have faster and more intense electrochemical activity. The differences in oscillation patterns across channels likely show how proteinoid‐algae interact in different ways. This could be due to local chemical environments, properties of the electrode‐sample interface, or self‐organizing behavior in the mixture (**Figure** **10**).

**Table 4:** Oscillation stats for the algae and Glu‐Phe proteinoid mix were taken from eight channels, ChA to ChH. The table shows the average amplitude (mV), average period (s), and the count of peaks for each channel. Data came from voltage recordings of the mixture. We found peaks and troughs using these thresholds: a minimum height of 10mV, a prominence of 5mV, and a minimum separation of 100s. This separation is equal to 250 samples at a sampling rate of 2.5Hz. Amplitudes show the differences from peak to trough in oscillation cycles. Periods measure the time between each peak. These metrics show how the algae‐GluPhe system oscillates. This may hint at dynamic interactions in electrochemistry.

Channel	Average amplitude [mV]	Average period [s]	Number of peaks
ChA [mV]	25.49	5471.01	32
ChB [mV]	33.75	3915.16	45
ChC [mV]	83.50	350.49	493
ChD [mV]	83.37	251.92	627
ChE [mV]	34.24	1447.43	120
ChF [mV]	191.42	403.78	396
ChG [mV]	33.72	4482.76	39
ChH [mV]	29.32	1930.98	89

The oscillatory behavior of pure Emilianiahuxleyi algae (Table 3) and the algae‐GluPhe proteinoid mixture (Table 4) shows clear differences in their electrical activities. These differences appear in all measured parameters. The pure algae sample has steady amplitude ranges of 35.94 to 80.94 mV across all channels. ChD and ChE show the highest amplitudes, ≈80 mV. The algae‐GluPhe mixture shows much more variability. ChF reaches 191.42 mV, which is over double the highest amplitude found in pure algae. This big increase shows that Glu:Phe proteinoids boost electrochemical activity in certain areas. Some channels (ChA, ChB, ChG, ChH) in the mixture show lower amplitudes than pure algae. This suggests that the proteinoids might inhibit some areas while boosting oscillations in others. The period measurements reveal even more striking differences. In pure algae, most channels show short periods, ranging from 299.88 to 929.53 s. This suggests a regular oscillatory behavior, except for ChD. Glu:Phe proteinoids change this pattern a lot. They usually extend the time in most channels. This is especially true for ChA, ChB, and ChG, where times rise to over 3900 s. This represents a 5–15 fold increase in oscillation periods for these channels. ChC and ChD in the mixture have very short periods, 350.49 and 251.92s. These are similar to the shortest periods found in pure algae. Proteinoids create complex spatiotemporal dynamics in the system. This affects oscillatory periods in two ways. The number of peaks detected further illustrates the altered dynamics. The pure algae sample shows a relatively even distribution of peaks across channels (185–480, with ChD as an outlier at 48). The mixture shows a very polarized distribution. ChC and ChD have high peak counts of 493 and 627. In contrast, other channels, like ChA, show much less activity, with only 32 peaks. This shift in oscillatory events shows that proteinoids create areas with strong, quick oscillations. At the same time, they reduce oscillations in other regions. These results show that Glu:Phe proteinoids change how the algae culture behaves electrically. This creates a more varied system with clear differences in both amplitude and frequency. The extreme values in the mixture, like ChF's amplitude and ChC/ChD's peak counts, suggest new properties. These may show more complex information processing.

To model the spiking behavior of these systems, a sinusoidal function with a damping term can be used to describe the potential V(t) as

(8) $$V\left(t\right)=A\sin\left(\frac{2\pi t}{T}\right)e^{-\alpha t}$$

where A is the average amplitude, T is the average period, and α is a damping factor. For pure algae, using ChE (amplitude 80.94 mV, period 724.00 s, 239 peaks), the equation is

(9) $$V_{\text{ChE}}\left(t\right)=80.94\sin\left(\frac{2\pi t}{724.00}\right)e^{-1.29\times 10^{-6}t}$$

with a small damping factor reflecting sustained oscillations over 48 h. For the algae‐Glu:Phe mixture, using ChF (amplitude 191.42 mV, period 403.78 s, 396 peaks), the equation is

(10) $$V_{\text{ChF}}\left(t\right)=191.42\sin\left(\frac{2\pi t}{403.78}\right)e^{-1.0\times 10^{-6}t}$$

Higher amplitude and shorter period show more frequent spiking. This happens with a slightly lower damping factor because of the increased oscillation frequency. These equations show a simpler view of the periodic spiking. They highlight the main differences in amplitude and period between the two systems.

This **Figure** **13** shows heatmaps of pairwise Pearson correlation coefficients. It compares the oscillatory signals from all channels (ChA to ChH). The left side displays data for pure *Emiliania huxleyi* algae. The right side shows results for the algae‐Glu:Phe proteinoid mixture. These correlations were calculated over a 10 000 s period. Color intensity shows the correlation value, ranging from –0.2 to 1.0. Red means a strong positive correlation, while blue indicates a weak or negative correlation. For pure algae, channels with similar periods show high correlations. For example, ChC and ChF have a correlation of 0.99 with periods of 543.99 and 563.72 s. ChE and ChH also show a strong correlation of 0.98, with periods of 724.00 and 647.16 s. This reflects synchronization, which supports coordinated dynamics in Boolean gate operations. An example is the OR gate frequency of 0.564 (Figure 15). Channels with disparate periods, such as ChB (299.88 s) and ChD (3611.79 s), show low correlations ( 0.10), indicating independent oscillatory behavior. The algae‐Glu:Phe mixture shows lower overall correlations. This is because it spans a wider period range (251.92–5471.01 s). Most values fall below 0.5, like ChA and ChD, which have values of 0.05 at periods of 5471.01 and 251.92 s. However, channels with closer periods, like ChC and ChF, have higher correlations, reaching 0.95 at periods of 350.49 and 403.78 s. This reduced synchronization matches the mixture's different oscillatory dynamics. It may also be affected by its mixed morphology. For example, the fibrous structures in Figure 5c could lead to varied signal generation. The pure algae shows higher correlations. This suggests it can perform synchronized computations better. In contrast, the mixture has lower correlations. This means it can respond independently and diversely, which may boost its performance in inverse logic gates, like NAND (0.883, Figure 15).

The observed electrical oscillations and their connection to light cycles may reflect this culture's unique behavior, not typical species responses. The patterns align with expected reactions to light cycles. However, without biological replicates, we can't confidently link these signals to species behavior. Future studies should include multiple biological replicates to separate random variation from real physiological responses. Also, the culturing conditions, like low‐nutrient media and temperatures between 15 and 20 °C, likely caused physiological stress. Previous studies on *E. huxleyi* have documented this stress,^[^ ^92^, ^107^, ^108^ ^]^ which could confuse the interpretation of these electrical signals.

### 3.7 Boolean Logic Implementation in Algae‐Proteinoid Networks

It's important to clarify that the Boolean operations presented here are derived from processing biological signals — they do not reflect the biological system's own computing abilities. We apply threshold functions to oscillatory patterns generated by biological components in order to construct logic gates. This distinction is crucial: conventional neuromorphic systems can process information and adapt autonomously, whereas our current system requires external input to interpret signals for computational use. True biohybrid computing must demonstrate the ability to alter its oscillatory behavior based on training or environmental feedback. We have not yet shown this capability in our current work. Nonetheless, the reliable generation of oscillatory signals that correspond to binary states represents an essential step toward functional bioelectronic computing.

The pure *Emiliania huxleyi* algae show oscillatory behavior. This, along with the algae‐Glu:Phe proteinoid mixture, allows for Boolean logic gates. This connection highlights their potential in unconventional computing. It links biological dynamics to traditional computing methods. **Figure** **14** shows how AND, OR, NOT, and XOR gates work. It uses binary signals from channels ChA and ChB over 10 000 s. Each system has its own unique oscillation patterns. The binary inputs come from a 50 mV threshold applied to the oscillatory signals. These signals are damped sinusoidal functions, based on average amplitudes and periods of the channels. In the pure algae system (see Figure 14a), the ChA signal has an average amplitude of 35.94 mV and a period of 929.53 s. It often drops below the threshold, resulting in a binary 0. In contrast, the ChB signal, with an average amplitude of 50.41 mV and a period of 299.88 s, usually exceeds the threshold, giving a binary 1. This amplitude difference creates a varied input pattern. In the top two panels, ChB has a higher amplitude and shorter period. This leads to more frequent changes between 0 and 1.

The Boolean gate outputs for the pure algae system reflect the logical operations applied to these binary inputs. The AND gate output in the third panel is 1 only when ChA and ChB signals exceed the threshold at the same time. This happens less often because ChA has a lower amplitude. As a result, high states occur ≈20% of the time. The OR gate in the fourth panel outputs a 1 when at least one input is 1. This results in high states ≈60% of the time, mainly due to ChB's strong signal. The NOT gate, applied to ChA, inverts its binary signal, showing a high output when ChA's signal is below 50 mV, which is most of the time given its amplitude. The XOR gate gives a 1 when the inputs are different. This matches the common mismatches between ChA and ChB. So, it produces a high output for ≈50% of the time. The results show that the pure algae system can mimic Boolean logic. It has a moderate amplitude range of 35.94–80.94 mV and diverse periods from 299.88 to 3611.79 s. ChB's 480 peaks lead to quicker changes in the binary signals.

Figure 14b shows the algae‐Glu:Phe mixture. It has a wider amplitude range, from 25.49 to 191.42 mV. The average period is longer too, at 2194.19 s. This reflects how the proteinoids affect the system's dynamics. The ChA signal (25.49 mV, 5471.01 s) hardly goes over the 50 mV threshold. This results in mostly a 0 binary input. ChB (33.75 mV, 3915.16 s) also finds it hard to cross the threshold. This means it has fewer transitions than the pure algae system. The AND gate outputs 1 only when both signals are above the threshold. This happens rarely, resulting in high states less than 10% of the time. The OR gate, however, benefits from the occasional high states in either channel, achieving a high output ≈30% of the time. The NOT gate on ChA usually outputs 1 because of ChA's low amplitude. The XOR gate shows the rare differences between the two inputs. It gives a high output for ≈20% of the time. The mixture has slower dynamics because it spends long periods in channels like ChA and ChG (≈4482.76 s). This leads to fewer transitions in the binary signals. As a result, it may slow down logical operations compared to the pure algae system. The mixture shows improved amplitude variability, especially in channels like ChF (191.42 mV). This shows a stronger ability to tell signals apart. That's helpful for uses needing clear binary outputs.

The comparison between the two systems highlights their distinct strengths in implementing Boolean gates. The pure algae system has frequent oscillations, averaging 256.25 peaks per channel. Channels like ChB show shorter periods. This leads to quicker and more dynamic binary transitions, which help with real‐time logical operations. The algae‐Glu:Phe mix has a higher average amplitude of 64.35 mV compared to 57.51 mV for pure algae. This means it sends stronger signals when the threshold is crossed. However, its longer periods result in fewer state changes, averaging 230.13 peaks per channel. This might slow down response times. These findings show the trade‐offs between speed and signal strength in biological computing systems. Pure algae offer fast dynamics, while the mixture boosts oscillatory strength for different logical outputs.

The oscillatory signals for channels ChA and ChB, denoted $V_{\text{ChA}}\left(t\right)$ and $V_{\text{ChB}}\left(t\right)$, are modeled as damped sinusoidal functions:

(11) $$V_{\text{Ch}}\left(t\right)=A_{\text{Ch}}\sin\left(\frac{2\pi t}{T_{\text{Ch}}}\right)e^{-\alpha t},\;\text{Ch}\in \left\{\text{ChA},\text{ChB}\right\},$$

where $A_{\text{Ch}}$ and $T_{\text{Ch}}$ are the channel‐specific average amplitude and period, and $\alpha =10^{-6}\;s^{-1}$ is the damping factor. The binary inputs $I_{\text{ChA}}\left(t\right)$ and $I_{\text{ChB}}\left(t\right)$ are derived by applying a threshold θ=50mV:

(12) $$I_{\text{Ch}}\left(t\right)=\left\{\begin{array}{cc} 1 & \text{if}\;V_{\text{Ch}}\left(t\right)\geq \theta ,\\ 0 & \text{if}\;V_{\text{Ch}}\left(t\right)<\theta , \end{array}\right.\;\text{Ch}\in \left\{\text{ChA},\text{ChB}\right\}.$$

The Boolean gate outputs are computed using the binary inputs $I_{\text{ChA}}\left(t\right)$ and $I_{\text{ChB}}\left(t\right)$. The following equations define the logical operations for all standard Boolean gates:

**AND Gate**: The output is 1 only if both inputs are 1.
(13) $$\begin{array}{ccc} O_{\text{AND}}\left(t\right) & = & I_{\text{ChA}}\left(t\right)\wedge I_{\text{ChB}}\left(t\right)\\ & = & \left\{\begin{array}{cc} 1 & \;\text{if}\;I_{\text{ChA}}\left(t\right)=1\;\text{and}\;I_{\text{ChB}}\left(t\right)=1,\\ 0 & \;\text{otherwise}. \end{array}\right. \end{array}$$
**OR Gate**: The output is 1 if at least one input is 1.
(14) $$\begin{array}{cc} O_{\text{OR}}\left(t\right) & =I_{\text{ChA}}\left(t\right)\vee I_{\text{ChB}}\left(t\right)=\\ & \left\{\begin{array}{cc} 1 & \text{if}\;I_{\text{ChA}}\left(t\right)=1\;\text{or}\;I_{\text{ChB}}\left(t\right)=1,\\ 0 & \text{otherwise}. \end{array}\right. \end{array}$$
**NOT Gate**: The output inverts the input (applied to ChA).
(15) $$\begin{array}{cc} O_{\text{NOT}}\left(t\right) & =\neg I_{\text{ChA}}\left(t\right)=\\ & \left\{\begin{array}{cc} 1 & \text{if}\;I_{\text{ChA}}\left(t\right)=0,\\ 0 & \text{if}\;I_{\text{ChA}}\left(t\right)=1. \end{array}\right. \end{array}$$
**XOR Gate**: The output is 1 if the inputs differ.
(16) $$\begin{array}{cc} O_{\text{XOR}}\left(t\right) & =I_{\text{ChA}}\left(t\right)⊕I_{\text{ChB}}\left(t\right)=\\ & \left\{\begin{array}{cc} 1 & \text{if}\;I_{\text{ChA}}\left(t\right)\neq I_{\text{ChB}}\left(t\right),\\ 0 & \text{if}\;I_{\text{ChA}}\left(t\right)=I_{\text{ChB}}\left(t\right). \end{array}\right. \end{array}$$
**NAND Gate**: The output is 0 only if both inputs are 1 (NOT of AND).
(17) $$\begin{array}{cc} O_{\text{NAND}}\left(t\right) & =\neg \left(I_{\text{ChA}}\left(t\right)\wedge I_{\text{ChB}}\left(t\right)\right)=\\ & \left\{\begin{array}{cc} 0 & \text{if}\;I_{\text{ChA}}\left(t\right)=1\;\text{and}\;I_{\text{ChB}}\left(t\right)=1,\\ 1 & \text{otherwise}. \end{array}\right. \end{array}$$
**NOR Gate**: The output is 0 if at least one input is 1 (NOT of OR).
(18) $$\begin{array}{cc} O_{\text{NOR}}\left(t\right) & =\neg \left(I_{\text{ChA}}\left(t\right)\vee I_{\text{ChB}}\left(t\right)\right)=\\ & \left\{\begin{array}{cc} 0 & \text{if}\;I_{\text{ChA}}\left(t\right)=1\;\text{or}\;I_{\text{ChB}}\left(t\right)=1,\\ 1 & \text{otherwise}. \end{array}\right. \end{array}$$
**XNOR Gate**: The output is 1 if the inputs are the same (NOT of XOR).
(19) $$\begin{array}{cc} O_{\text{XNOR}}\left(t\right) & =\neg \left(I_{\text{ChA}}\left(t\right)⊕I_{\text{ChB}}\left(t\right)\right)=\\ & \left\{\begin{array}{cc} 1 & \text{if}\;I_{\text{ChA}}\left(t\right)=I_{\text{ChB}}\left(t\right),\\ 0 & \text{if}\;I_{\text{ChA}}\left(t\right)\neq I_{\text{ChB}}\left(t\right). \end{array}\right. \end{array}$$

The Boolean gates use the oscillatory dynamics of pure *Emiliania huxleyi* algae and a Glu:Phe proteinoid mix. They work with binary inputs from channels ChA and ChB, as shown in Figure 14. Binary signals come from thresholding the oscillatory signals. This is done at half the average amplitude for each channel. For example, it's 17.97 mV for ChA in pure algae and 12.75 mV for ChA in the mixture. This process creates binary values of 0 or 1. The truth tables for the AND, OR, NOT, XOR, NAND, NOR, and XNOR gates are shown in Figure 16. They define the logical links between the inputs $I_{\text{ChA}}\left(t\right)$, $I_{\text{ChB}}\left(t\right)$, and the outputs $O_{\text{GATE}}\left(t\right)$.

Figure 16(a) shows the truth table for the AND gate, which outputs 1 only when both inputs are 1. In the pure algae system, this condition happens ≈25% of the time, as shown in **Figure** **15**. ChB has a higher amplitude of 50.41 mV, which often surpasses its threshold of 25.21 mV. In contrast, ChA, with an amplitude of 35.94 mV and a threshold of 17.97 mV, crosses less frequently. In the mixture, the AND gate's high state is rare, ≈5%. ChA (25.49 mV) and ChB (33.75 mV) both find it hard to reach their thresholds of 12.75 mV and 16.88 mV. This shows their slower dynamics, like ChA's long period of 5471.01 s. The OR gate, in **Figure** **16**b, outputs 1 when at least one input is 1, resulting in a higher frequency of high states (pure algae: 75%, mixture: 30%), as ChB in pure algae dominates the output. The NOT gate on ChA in Figure 16c flips the input. It gives a high output when ChA drops below its threshold. This happens often in the mixture, ≈70% of the time, because ChA has a low amplitude.

The XOR gate, seen in Figure 16d, gives an output of 1 when the inputs are not the same. This matches the observed frequencies: ≈50% for pure algae and ≈25% for the mixture. The pure algae system has more mismatched inputs because of ChB's quick oscillations, which happen every 299.88 s and create 480 peaks. The NAND gate (Figure 16e) and NOR gate (Figure 16f) act as the opposites of AND and OR. They produce high outputs when the AND and OR conditions fail. This leads to more frequent 1s than their counterparts. For example, the NAND frequency is ≈75% for pure algae and ≈95% for the mixture. The XNOR gate (Figure 16g) gives an output of 1 when the inputs match. This is the opposite of XOR. For pure algae, the expected frequency is ≈50%. For the mixture, it's ≈75%. These truth tables show logical operations. The frequency of each input combination reflects the unique oscillatory dynamics of the systems. Their morphological traits affect this, like how dense clustering in Figure 5a boosts the mixture's signal strength. After analyzing the Boolean gate outputs and frequencies (see Figures 14 and 15), Figure 16 shows the truth tables for all the gates. This formalizes the logical operations. The AND gate (Figure 16a) needs both inputs to be 1. This happens more often in the pure algae system because of ChB's dynamic signal. In contrast, the mixture's slower dynamics limit this condition. The OR, NOT, and XOR gates (Figure 16b–d) further illustrate the systems' logical behavior, with the mixture's NOT gate showing a higher frequency of 1s due to ChA's low amplitude. The extra gates (NAND, NOR, XNOR) offer a full range of logical operations. This enhances the system's flexibility in unique computing tasks.

### 3.8 pH and Temperature as Regulatory Parameters in Algae‐Proteinoid Electrochemical Dynamics

**Figure** **17** shows how temperature and pH affect the oscillatory dynamics of pure *Emiliania huxleyi* algae. This data covers a period of 46 381 s, or ≈12.9 h. The temperature drops quickly from 18.932 to 15.19 °C at ≈5000 s. Then, it rises to ≈17.5 °C by 20 000 s. This shows a slight increase of 0.116 °C, with a slope of 0.000003 °C s^−1^. The pH (bottom) rises quickly from 5.96 to 8.03 in the first 5000 s. This change is likely caused by photosynthesis. After that, it levels off ≈7.5, showing a slight linear trend with just a 0.003 pH change (slope: 0.000000 pH s^−1^). **Figure** **18** shows how temperature and pH change over time. It reveals big swings, like temperature shifts of ±3.0 °C and pH changes of ±1.5, at ≈5000 and 20 000 s. There are also smaller oscillations with periods of 300 to 600 s. These may be linked to metabolic cycles in the 12 h light and dark cycle. These environmental changes might relate to the algae's electrical activity. For example, ChB has a period of ≈299.88 s and 480 peaks (see Figure 14). This activity can affect how well the algae performs Boolean gate operations (see Figure 16). Stable environments may improve the reliability of direct gates, like the OR gate, which has a frequency of 0.564 (see Figure 15).

The pH oscillations observed in Figure 17 are driven by the interplay of photosynthetic and respiratory processes in the pure *Emiliania huxleyi* algae, which affect ${\mathrm{CO}}_{2}$ levels in the medium. During photosynthesis, primarily in the light phase of the 12h:12h cycle, algae consume ${\mathrm{CO}}_{2}$, reducing carbonic acid and increasing pH:

(20) $$6{\mathrm{CO}}_{2}+6H_{2}O+\mathrm{light}⟶C_{6}H_{1}2O_{6}+6O_{2}$$

The dissolved ${\mathrm{CO}}_{2}$ forms carbonic acid, which dissociates according to:

(21)

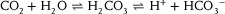




The consumption of ${\mathrm{CO}}_{2}$ during photosynthesis reduces $H_{2}{\mathrm{CO}}_{3}$ formation, decreasing 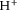 concentration and increasing pH, as defined by:

(22)

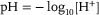

The rapid pH increase from 5.96 to 8.03 within the first 5000 s likely reflects a burst of photosynthetic activity, possibly triggered by the onset of the light phase.

During respiration, particularly in the dark phase or when photosynthesis slows,^[^ ^109^ ^]^ algae produce ${\mathrm{CO}}_{2}$, increasing $H_{2}{\mathrm{CO}}_{3}$ and 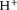 concentration, thus decreasing pH:

(23) $$C_{6}H_{1}2O_{6}+6O_{2}⟶6{\mathrm{CO}}_{2}+6H_{2}O+\mathrm{energy}$$

This shifts the equilibrium in Equation (21) to produce more $H_{2}{\mathrm{CO}}_{3}$ and 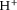, lowering pH. The stabilization of pH ≈7.5 after the initial rise suggests a buffering effect from the bicarbonate system in the medium:

(24)

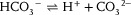




This buffer resists large pH changes by adjusting 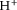 concentration, contributing to the smaller pH oscillations observed after 5000 s (e.g., ±0.1 deviations, Figure 18). These pH oscillations may influence the algae's electrical activity, as 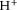 concentration affects membrane potentials and ion transport, potentially impacting the performance of Boolean gate operations (e.g., OR gate frequency: 0.564, Figure 15).

### 3.9 Environmental Tolerance and Adaptability of Algal Species for Biocomputing Applications

Algae species respond differently to temperature and pH. This shows their adaptations and how they function. These factors can affect their use in areas like unconventional computing. *Chlamydomonas reinhardtii* is a green alga often used to study photosynthesis and circadian rhythms.^[^ ^110^, ^111^ ^]^ It grows best in temperatures between 20and30 °C, with 25 °C being ideal. When temperatures rise above 35 °C, its photosynthesis drops because enzymes can break down. On the other hand, temperatures below 15 °C slow its metabolism, which lowers ${\mathrm{CO}}_{2}$ uptake and affects pH. *Chlamydomonas* likes a neutral to slightly alkaline pH, ≈ 7to8. In this range, it manages its internal pH well using proton pumps and bicarbonate uptake. Acidic conditions (like pH<6) can hurt photosynthesis. They disrupt the proton gradient in the thylakoid membrane. On the other hand, highly alkaline conditions (pH>9) can stress the alga. This stress can reduce growth and change gene expression. These responses suggest that *Chlamydomonas* can keep steady oscillations for computing tasks within its ideal temperature and pH. However, extreme conditions may disrupt its electrical activity.

*Ostreococcus tauri* ^[^ ^112^, ^113^ ^]^ is a tiny green alga that lives in the ocean. It responds differently to changes in temperature and pH because of its small size and floating living arrangement. It grows best at 18−22 °C, showing its fit for cooler ocean waters. It can handle a narrow range of 15−25 °C before feeling thermal stress. *Ostreococcus* has less chlorophyll and lower photosynthesis rates at temperatures over 25 °C. Below 15 °C, its cell division slows down. This affects ${\mathrm{CO}}_{2}$ use and pH control. *Ostreococcus* thrives in stable, slightly alkaline seawater with a pH of 8.0−8.3. It uses effective carbon concentrating mechanisms (CCMs) to take in bicarbonate (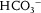). Acidic conditions (pH<7.5) can slow down its CCMs. This reduces photosynthetic efficiency and leads to smaller pH changes. On the other hand, pH levels above 8.5 may cause calcium carbonate to form in the ocean. This can stress the alga indirectly. For unconventional computing, *Ostreococcus*'s stable pH response in the ocean could help create steady electrical oscillations. However, its narrow temperature range may limit how well it adapts to changing environments, especially when compared to *Chlamydomonas*.

Diatoms, such as *Phaeodactylum tricornutum*,^[^ ^114^, ^115^ ^]^ thrive in both marine and freshwater. They handle changes in temperature and pH well. This resilience makes them strong choices for environmental monitoring and computing. *Phaeodactylum* grows best at 18−23 °C. It can handle a range from 5 to 30 °C. However, temperatures over 30 °C lead to oxidative stress and less silica in its cell walls. On the other hand, when temperatures drop below 10 °C, growth slows down but lipid production increases. Photosynthesis affects pH. The best pH level is between 7.5and8.5. When plants take in ${\mathrm{CO}}_{2}$, the pH rises (like from 7.5to8.5). But respiration lowers the pH. In acidic conditions (pH<6.5), *Phaeodactylum* upregulates CCMs to maintain carbon fixation, but prolonged exposure can reduce growth rates. In very alkaline conditions (pH>9), it might face nutrient limits. This can impact metabolic changes. The diatom can keep pH levels steady even when temperatures change. This means it might handle electrical tasks better than *Chlamydomonas* and *Ostreococcus* in shifting environments. Its strong silica cell walls, like those of pure algae, help it generate stable signals.

**Figure 19:**
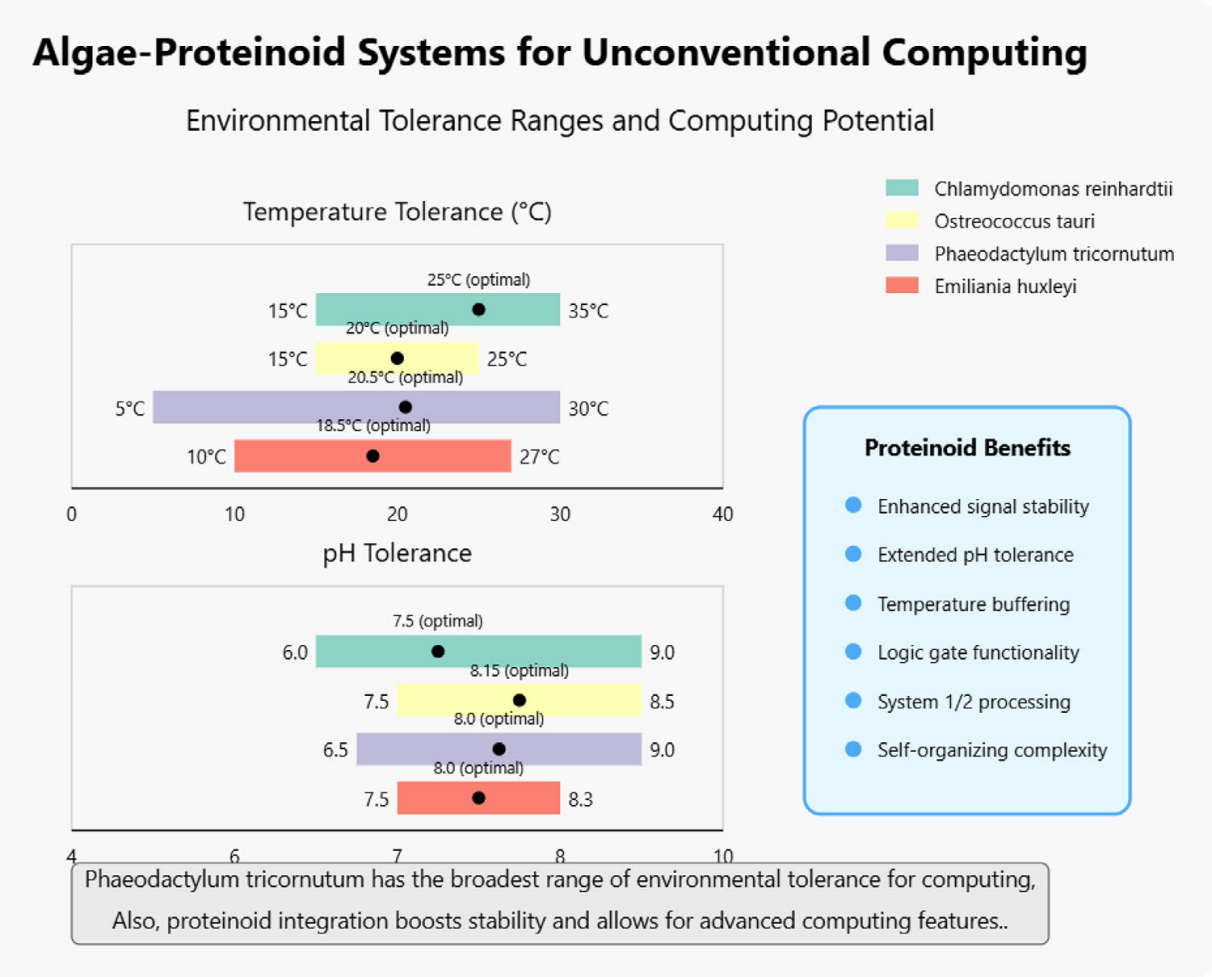
Environmental tolerance ranges of four algae species (*Chlamydomonas reinhardtii*, *Ostreococcus tauri*, *Phaeodactylum tricornutum*, and *Emiliania huxleyi*) for unconventional computing applications. The upper panel shows temperature tolerance ranges (°C). The black circles mark the optimal growth temperatures. *P. tricornutum* exhibits the widest thermal tolerance (5–30°C), while *O. tauri* shows the narrowest range (15–25°C). The middle panel shows pH tolerance. *C. reinhardtii* and *P. tricornutum* have the widest ranges, 6.0–9.0 and 6.5–9.0, respectively. In contrast, *E. huxleyi* has a narrower range of 7.5–8.3. The integration of proteinoids with algae confers multiple benefits (right panel): enhanced signal stability, extended environmental tolerance, logical operation functionality, and emergent computational capabilities including System 1/2 processing. Among the species studied, *P. tricornutum* shows the best environmental strength for biocomputing. This is especially true when combined with proteinoid structures.

**Figure** **19** shows the environmental tolerance of four algal species. These species may have uses in unconventional biocomputing. The analysis shows that different species have varied ranges for temperature and pH tolerance. *Phaeodactylum tricornutum* shows the best thermal adaptability, thriving from 5 to 30 °C. This range surpasses *Chlamydomonas reinhardtii*, which can live between 15 and 35 °C, even though it has a higher upper limit. The wide temperature range of *P. tricornutum* is a key benefit for biocomputing. It can work well even when conditions change. pH tolerance data show that both *P. tricornutum* and *C. reinhardtii* work well across a wide pH range (6.5–9.0). In contrast, *Emiliania huxleyi* has a much narrower pH range (7.5–8.3). Combining proteinoids with algal systems brings many benefits. First, it improves signal stability. Second, it boosts environmental tolerance. Finally, it adds new features like logical gate operations and dual‐process computing. These findings show that *P. tricornutum*‐proteinoid hybrid systems are strong. They combine environmental strength with great computing power. This makes them good choices for biocomputing projects, especially those needing to withstand changing conditions.

### 3.10 Biohybrid Computing: Proteinoid‐Algae Interfaces and Performance Analysis

Sidney Fox was a pioneering biochemist. In the 1950s, he synthesized proteinoids. He saw them as important for studying life's origins and how cells work. Fox called them “proto‐neurons” because they could mimic electrical activity like neurons. Fox noticed that proteinoid microspheres can form when amino acids are heated in prebiotic conditions. They show membrane potentials and can create action potential‐like spikes when stimulated. This behavior is like that of biological neurons. He suggested that these properties made proteinoids possible precursors to early cellular systems. They could process basic information by responding to environmental changes, like pH, temperature, or chemical gradients. This proto‐neuronal behavior comes from microspheres forming ion‐selective membranes. They help with charge transfer, letting the spheres process information like neurons. They encode inputs by changing spike frequency or amplitude. In unconventional info processing, proteinoids can do computations. They perform logical operations and recognize patterns. Their spiking patterns map to binary states. This helps simulate neural networks. So, they connect prebiotic chemistry with modern bio‐inspired computing systems.

Algae and proteinoids can work well together. This combination boosts their use in unconventional computing. Algae's ability to photosynthesize and proteinoids' electrical response create a powerful system. Algae such as *Chlamydomonas reinhardtii* and *Phaeodactylum tricornutum* contain many proteins and bioactive compounds. Their metabolic activity can change with environmental factors, such as temperature and pH. This, in turn, can affect how proteinoids behave. For example, algae can change the local chemical environment through photosynthesis. This process includes consuming ${\mathrm{CO}}_{2}$ and producing $O_{2}$. These changes affect the conductivity and spiking patterns of proteinoid microspheres. Studies show that when proteinoids mix with chondroitin sulfate clusters, their electrical activity changes.^[^ ^64^ ^]^ This combination might allow computing systems to adapt to their environment. For example, they could respond to changes in light and pH. This could mimic how synaptic plasticity works in neural networks. Additionally, algae's ability to produce therapeutic proteins (e.g., in *Schizochytrium sp*.)—vaccine antigens^[^ ^116^ ^]^—could work with proteinoids' ability to process information. This combination may lead to bio‐computational devices that sense, compute, and deliver therapies. Such advancements could benefit sustainable biotechnology and brain‐inspired computing.

This study shows how proteinoids can work with *Emiliania huxleyi* algae. Together, they create biohybrid systems for unconventional computing. The algae use photosynthesis, while the proteinoids act like neurons. This combination can perform logical operations. The pH changes we observed come from photosynthesis and respiration (Equations (20), (21), (22), (23), (24)). Temperature changes occur due to metabolic heat and heat exchange with the environment. These factors show how the environment affects the algae's electrical activity. For example, in channels like ChB, we noted a 299.88 s period with 480 peaks. These oscillations relate to how well the systems perform in Boolean gate operations (Figure 14). The pure algae often cross thresholds, boosting direct gate frequencies (e.g., OR: 0.564, Figure 15). In contrast, the algae‐Glu:Phe mixture shows varied dynamics that help with inverse gates (e.g., NAND: 0.883). The channel correlation heatmaps (Figure 13) show how pure algae act in sync. In contrast, the mixture responds independently due to their different morphologies (Figure 5). This indicates that both environmental and structural factors are key to their behavior. Environmental changes show the promise of bio‐inspired materials for sustainable computing. They can help create dynamic responses in biohybrid systems. The pH and temperature changes, with periods of ≈300 to 600 s, match ChB's electrical oscillation. This suggests that environmental factors can impact the algae's computational reliability. This is evident in the error rates, such as the OR of 0.10 (see Figure 15b). This interplay shows we need materials that work well in changing conditions. Various unconventional computing materials tackle this challenge. Table 5 compares proteinoids, fungi (Basidiomycetes), and slime molds (*Physarum polycephalum*). It shows their pros and cons. Proteinoids offer biocompatibility and neuron‐like spiking but face scalability issues due to slow response times, while fungi provide resilient, self‐repairing networks but are limited by low‐frequency signaling. Slime molds excel in pathfinding, yet their dynamic geometry hinders device stability. These materials help global eco‐friendly tech efforts because they are biodegradable and use little energy. However, their slow processing speeds and instability indicate that we need hybrid methods. Combining them with traditional electronics will better unlock their potential in next‐gen computing. The algae‐proteinoid system in this study shows the potential and challenges of biohybrid computing. It offers a sustainable way to connect biology and computing. The algae's photosynthetic changes and the proteinoids' electrical spikes work together to respond to environmental changes. This is shown through the Boolean gate operations (Figure 14) and channel correlations (Figure 13). However, the mixture has slower dynamics and higher error rates, like the AND operation at 0.17 (Figure 15). This shows we need to improve stability and response times. Environmental changes (Figures 17 and 18) show that better growth conditions can increase reliability. Stabilizing pH and temperature may help. This could be due to the algae's uniform shape (Figure 5), which supports steady metabolic activity. Future research directions should prioritize the development of sophisticated hybrid interfaces that integrate algae‐proteinoid systems with traditional electronic components, addressing fundamental challenges related to scalability, stability, and long‐term performance reliability. These investigations should encompass applications in neuromorphic computing and environmental sensing technologies, ultimately advancing the field of sustainable, bio‐inspired computational systems.

**Table 5:** This table compares unconventional computing materials: proteinoids, fungi (Basidiomycetes), and slime molds (*Physarum polycephalum*). It shows their strengths and weaknesses. Proteinoids are biocompatible and mimic neuron spiking, making them great for biohybrid systems. However, their slow response times and instability hold back scalability. Fungi have strong, self‐repairing networks that could be used for microfluidic computing. However, their low‐frequency signaling and specific environmental needs make practical use difficult. Slime molds are great at finding paths and adapting. They can even mimic efficient transport networks. However, their changing shape and slow processing limit their use to experiments. These materials help global efforts for eco‐friendly technologies. They provide biodegradable, low‐energy options for traditional semiconductors. However, their slow processing speeds and lack of long‐term stability show we need hybrid approaches. Combining them with conventional electronics can unlock their full potential in next‐generation computing.

Material	Advantages	Limitations
Proteinoids^[^ ^117^ ^]^	Self‐assemble into microspheres that behave like neurons with spiking; Biocompatible and biodegradable; Mimic biological functions, such as electrical signaling; Process with low energy.	Limited structural stability over time; Slow response times, often taking minutes; Hard to scale for complex computations; No standard interfaces with regular electronics.
Fungi (Basidiomycetes)^[^ ^118^, ^119^ ^]^	Large mycelium networks enable parallel processing; They resist environmental stress, like radiation; They can self‐repair and are fault‐tolerant; They use low energy. They have potential in microfluidic computing.	Slow electrical signaling (low‐frequency oscillations, e.g., 0.1–1 Hz); Complex shapes make precise calculations tricky; It needs humid conditions; Long‐term stability of the device is uncertain.
Slime Molds (Physarum polycephalum)^[^ ^120^, ^121^, ^122^ ^]^	Efficient pathfinding, like finding the shortest paths or optimizing networks; Adapts to stimuli, such as light and chemicals; Solves computational geometry problems; Fault‐tolerant because of emergent behavior.	Changing network geometry affects device stability over time; Computation can be slow, taking hours for complex tasks; Devices are limited to lab settings and are sensitive to environmental factors like dryness.

## 4 Conclusion

We studied bioelectronic signal processing systems made of proteinoid microspheres and algae. This work shows a great way to create sustainable and biocompatible platforms for basic computational tasks. The L‐Glu:L‐Phe proteinoid microspheres are combined with *Emiliania huxleyi* algae to form a composite material. The fabrication process yields specialized materials characterized by unique morphological features and intriguing electrical oscillation patterns that are essential for computational operations. Our current system leverages these oscillations to implement Boolean logic functions through sophisticated post‐acquisition processing of the generated biological signals, rather than relying on autonomous computing methods. We designate this as a biohybrid system based on the integrated biological and synthetic materials employed, representing a significant advancement toward genuine biohybrid computing while acknowledging that it does not yet constitute a complete platform with autonomous learning capabilities. The algae‐proteinoid mix works better in inverse gate operations. Environmental factors, such as temperature and pH, can present challenges and opportunities for these bioelectronic systems. Environmental changes can affect signal processing performance, but they also allow for adaptive responses. This adaptability can be useful for applications that need to respond to different contexts. Comparing with other algal species shows that choosing the right species and modifying proteinoids can improve reliability. Such modifications could also widen the range of conditions in which they can operate. Neurone‐like spiking in these algae‐proteinoid systems links big questions about chemical evolution to practical uses in unconventional computing. We show a new way to create bioelectronic signal processing platforms. This method uses proteinoid‐algae interfaces. They blend the flexibility of living systems with the programming performance of traditional computers. Future work must specifically address the limitations of our current approach by developing mechanisms for feedback and adaptation within these biological systems, potentially enabling true learning capabilities. Future research should aim to improve system stability. It should also expand the range of computations beyond simple logic gates. Finding ways to combine biological and electronic parts is crucial. This is vital for making true hybrid bioelectronic systems that can demonstrate autonomous information processing rather than requiring external interpretation of signals. These advancements may lead to significant changes in environmental sensing, biomedical diagnostics, and sustainable computing. These fields will look to nature for inspiration in processing information.

## Conflict of Interest

The authors declare no conflict of interest.

## Data Availability

The data that support the findings of this study are openly available in The data are available here: https://zenodo.org/records/15163989 at The data are available here: https://zenodo.org/records/15163989 , reference number 15163989.

## References

[1] A. Adamatzky , T. Schubert , Mater. Today 2014, 17, 86.

[2] B. Wijerathne , T. Liao , X. Jiang , J. Zhou , Z. Sun , Mater. Futures 2025, 4, 012301.

[3] S. W. Fox , T. Nakashima , BioSystems 1980, 12, 155. 7397322 10.1016/0303-2647(80)90013-1

[4] L. Valentini , Mater. Lett. 2015, 148, 204.

[5] P. Mougkogiannis , Printable Chemical Sensors Based on Organic Field Effect Transistors, The University of Manchester, Manchester, UK, 2021.

[6] M. Irimia‐Vladu , Chem. Soc. Rev. 2014, 43, 588. 24121237 10.1039/c3cs60235d

[7] S. Sahu , A. Kaur , G. Singh , S. K. Arya , Energy Ecol. Environ. 2024, 1.

[8] M. Berggren , A. Richter‐Dahlfors , Adv. Mater. 2007, 19, 3201.

[9] K. Balasubramanian , M. Burghard , Anal. Bioanal. Chem. 2006, 385, 452. 16568294 10.1007/s00216-006-0314-8

[10] S.‐W. Hwang , H. Tao , D.‐H. Kim , H. Cheng , J.‐K. Song , E. Rill , M. A. Brenckle , B. Panilaitis , S. M. Won , Y.‐S. Kim , Y. M. Song , K. J. Yu , A. A. Ameen , R. Li , Y. Su , M. Yang , D. L. Kaplan , M. R. Zakin , M. J. Slepian , Y. Huang , F. G. Omenetto , J. A. Rogers , Science 2012, 337, 1640. 23019646 10.1126/science.1226325 PMC3786576

[11] T. Someya , Z. Bao , G. G. Malliaras , Nature 2016, 540, 379. 27974769 10.1038/nature21004

[12] J. Rivnay , S. Inal , A. Salleo , R. M. Owens , M. Berggren , G. G. Malliaras , Nat. Rev. Mater. 2018, 3, 1.

[13] R. Feiner , T. Dvir , Nat. Rev. Mater. 2017, 3, 1.

[14] P. Mougkogiannis , A. Adamatzky , ACS Omega 2025. 10.1021/acsomega.4c10571 PMC11923683 40124033

[15] P. Mougkogiannis , A. Nikolaidou , A. Adamatzky , ACS omega 2024, 9, 45789. 39583677 10.1021/acsomega.4c03546 PMC11579727

[16] P. Mougkogiannis , A. Adamatzky , Smart Mater. Struct. 2024, 34, 015054.

[17] P. Mougkogiannis , A. Adamatzky , Langmuir 2024, 40, 12649. 38837748 10.1021/acs.langmuir.4c01107 PMC11191697

[18] S. W. Fox , Geochim. Cosmochim. Acta 1995, 59, 1213. 11540049 10.1016/0016-7037(95)00037-z

[19] P. Mougkogiannis , A. Adamatzky , ACS omega 2023, 8, 35417. 37780014 10.1021/acsomega.3c05670 PMC10536103

[20] P. Mougkogiannis , A. Adamatzky , ACS omega 2024, 9, 51098. 39758676 10.1021/acsomega.4c06401 PMC11696383

[21] P. Mougkogiannis , A. Adamatzky , ACS Appl. Bio Mater. 2025, 8, 854. 10.1021/acsabm.4c01678 PMC11752506 39779286

[22] P. Mougkogiannis , A. Adamatzky , BioSystems 2024, 237, 105175. 38460836 10.1016/j.biosystems.2024.105175

[23] P. Mougkogiannis , A. Adamatzky , ACS Omega 2025, 10, 5016. 39959035 10.1021/acsomega.4c10790 PMC11822715

[24] L. Jia , Y. Li , A. Ren , T. Xiang , S. Zhou , ACS Appl. Mater. Interfaces 2024, 16, 32887. 38904545 10.1021/acsami.4c05663

[25] A. Nayak , I. Satpathy , in Revolutionizing Automated Waste Treatment Systems: IoT and Bioelectronics, IGI Global, Pennsylvania, USA 2024, pp. 221–236.

[26] C. Lei , Q. Li , W. Chen , G. Yu , Adv. Mater. 2025, 2419906. 10.1002/adma.202419906 39924805

[27] J.‐W. Choi , Y.‐S. Nam , W. H. Lee , Curr. Appl. Phys. 2002, 2, 79.

[28] L. Zhang , J. Lu , T. Waigh , Adv. Colloid Interface Sci. 2021, 287, 102319. 33248339 10.1016/j.cis.2020.102319

[29] M. Zubair , S. Hussain , A. Hussain , M. E. Akram , S. Shahzad , Z. Rauf , M. Mujahid , A. Ullah , Biomater. Sci. 2025, 13, 130. 10.1039/d4bm01099j 39569610

[30] S. Tansaz , A. R. Boccaccini , J. Biomed. Mater. Res., Part A 2016, 104, 553. 10.1002/jbm.a.35569 26402327

[31] Y. Pan , H. Zhao , W. Huang , S. Liu , Y. Qi , Y. Huang , Adv. Healthcare Mater. 2025, 14, 2404405. 10.1002/adhm.202404405 39778029

[32] Y. Zou , Y. Zhong , H. Li , F. Ding , X. Shi , Curr. Med. Chem. 2020, 27, 2610. 31830879 10.2174/0929867326666191212163955

[33] K. Z. Lee , J. Jeon , B. Jiang , S. V. Subramani , J. Li , F. Zhang , Molecules 2023, 28, 4988. 37446650 10.3390/molecules28134988 PMC10343515

[34] S. Chu , A. L. Wang , A. Bhattacharya , J. K. Montclare , Prog. Biomed. Eng. 2021, 4, 012003. 10.1088/2516-1091/ac2841 PMC8691744 34950852

[35] M. Santos , S. Serrano‐Dúcar , J. González‐Valdivieso , R. Vallejo , A. Girotti , P. Cuadrado , F. J. Arias , Curr. Med. Chem. 2019, 26, 7117. 29737250 10.2174/0929867325666180508094637

[36] E. Troy , M. A. Tilbury , A. M. Power , J. G. Wall , Polymers 2021, 13, 3321. 34641137 10.3390/polym13193321 PMC8513057

[37] E. Zdraveva , V. Gaurina Srček , K. Kraljić , D. Škevin , I. Slivac , M. Obranović , Polymers 2023, 15, 2684. 37376328 10.3390/polym15122684 PMC10301402

[38] S. Gopalakrishnan , J. Xu , F. Zhong , V. M. Rotello , Adv. Sustainable Syst. 2021, 5, 2000167. 33709022 10.1002/adsu.202000167 PMC7942017

[39] H. Lee , Y. Won , J. H. Oh , J. Polym. Sci. 2022, 60, 348.

[40] I. Krauhausen , C.‐T. Coen , S. Spolaor , P. Gkoupidenis , Y. van de Burgt , Adv. Funct. Mater. 2024, 34, 2307729.

[41] P. Belleri , J. Pons i Tarrés , I. McCulloch , P. W. Blom , Z. M. Kovács‐Vajna , P. Gkoupidenis , F. Torricelli , Nat. Commun. 2024, 15, 5350. 38914568 10.1038/s41467-024-49668-1 PMC11196688

[42] S. Yang , J. Wang , B. Deng , M. R. Azghadi , B. Linares‐Barranco , IEEE Trans. Neural Netw. Learn. Syst. 2021, 33, 7126. 10.1109/TNNLS.2021.3084250 34115596

[43] R. Patton , C. Schuman , S. Kulkarni , M. Parsa , J. P. Mitchell , N. Q. Haas , C. Stahl , S. Paulissen , P. Date , T. Potok , S. Snyder , in International Conference on Neuromorphic Systems 2021, Association for Computing Machinery, New York 2021, pp. 1–5.

[44] N. Qiao , H. Mostafa , F. Corradi , M. Osswald , F. Stefanini , D. Sumislawska , G. Indiveri , Front. Neurosci. 2015, 9, 141. 25972778 10.3389/fnins.2015.00141 PMC4413675

[45] F. Zhang , Z. Li , C. Chen , H. Luan , R. H. Fang , L. Zhang , J. Wang , Adv. Mater. 2024, 36, 2303714. 10.1002/adma.202303714 PMC10799182 37471001

[46] A. T. Przybylski , S. W. Fox , Appl. Biochem. Biotechnol. 1984, 10, 301. 11536591 10.1007/BF02783764

[47] G. Vaughan , A. T. Przybylski , S. W. Fox , BioSystems 1987, 20, 219. 3620604 10.1016/0303-2647(87)90028-1

[48] A. T. Przybylski , BioSystems 1985, 17, 281. 4052590 10.1016/0303-2647(85)90044-9

[49] P. Mougkogiannis , N. R. Kheirabadi , A. Adamatzky , New J. Chem. 2024, 48, 17650.

[50] S. W. Fox , (No Title) 1988.

[51] S. W. Fox , in Evolution of Information Processing Systems: An Interdisciplinary Approach for a New Understanding of Nature and Society, Springer, Berlin 1992, pp. 203–228.

[52] A. Adamatzky , L. Bull , B. D. L. Costello , Unconventional computing 2007, Luniver Press, Bristol, England 2007.

[53] M. Irimia‐Vladu , E. D. Głowacki , P. A. Troshin , G. Schwabegger , L. Leonat , D. K. Susarova , O. Krystal , M. Ullah , Y. Kanbur , M. A. Bodea , V. F. Razumov , H. Sitter , S. Bauer , N. S. Sariciftci , Adv. Mater. 2012, 24, 375. 22109816 10.1002/adma.201102619

[54] M. J. Tan , C. Owh , P. L. Chee , A. K. K. Kyaw , D. Kai , X. J. Loh , J. Mater. Chem. C 2016, 4, 5531.

[55] M. Al‐Mossawi , H. Warren , P. J. Molino , P. Calvert , M. in het Panhuis , Mater. Adv. 2021, 2, 1369.

[56] S. W. Fox , K. Harada , J. Am. Chem. Soc. 1960, 82, 3745.

[57] A. T. Przybylski , W. P. Stratten , R. M. Syren , S. W. Fox , Naturwissenschaften 1982, 69, 561. 7162535 10.1007/BF00396351

[58] P. Mougkogiannis , A. Adamatzky , Biomimetics 2024, 9, 380. 39056821 10.3390/biomimetics9070380 PMC11275190

[59] D. Schulze‐Makuch , L. N. Irwin , Naturwissenschaften 2006, 93, 155. 16525788 10.1007/s00114-005-0078-6

[60] M. A. Boden , Int. J. Astrobiol. 2003, 2, 121.

[61] B. Damer , D. Deamer , Astrobiology 2020, 20, 429. 31841362 10.1089/ast.2019.2045 PMC7133448

[62] T. West , V. Sojo , A. Pomiankowski , N. Lane , Philos. Trans. R. Soc. B 2017, 372, 20160419. 10.1098/rstb.2016.0419 PMC5665807 29061892

[63] V. Kompanichenko , Int. J. Astrobiol. 2008, 7, 27.

[64] P. Mougkogiannis , A. Adamatzky , Plos one 2024, 19, e0313077. 39630635 10.1371/journal.pone.0313077 PMC11616837

[65] P. Mougkogiannis , A. Adamatzky , Mater. Today Bio 2024, 25, 100989. 10.1016/j.mtbio.2024.100989 PMC10879779 38384791

[66] P. Mougkogiannis , A. Adamatzky , Mater. Des. 2023, 236, 112460.

[67] M. Tilli , M. Paulasto‐Kröckel , M. Petzold , H. Theuss , T. Motooka , V. Lindroos , Handbook of silicon based MEMS materials and technologies, Elsevier, Amsterdam 2020.

[68] M. Moustakas , Materials 2021, 14, 549. 33498822 10.3390/ma14030549 PMC7866148

[69] H. V. Westerhoff , P. R. Jensen , J. L. Snoep , B. N. Kholodenko , Thermochim. Acta 1998, 309, 111.

[70] M. Levin , Trends Cell Biol. 2007, 17, 261. 17498955 10.1016/j.tcb.2007.04.007

[71] A. Adamatzky , Advances in Unconventional Computing: Volume 1: Theory, vol. 22, Springer, Berlin 2016.

[72] P. Mougkogiannis , A. Adamatzky , R. Soc. Open Sci. 2023, 10, 230936. 37830018 10.1098/rsos.230936 PMC10565364

[73] P. Mougkogiannis , A. Adamatzky , ACS omega 2025. 10.1021/acsomega.4c10571 PMC11923683 40124033

[74] N. Purali , B. Rydqvist , J. Neurophysiol. 1998, 80, 2121. 9772266 10.1152/jn.1998.80.4.2121

[75] F.‐L. Ng , K. Yunus , V. Periasamy , A. C. Fisher , S.‐M. Phang , Sci. Rep. 2017, 7, 16237. 29176639 10.1038/s41598-017-16530-y PMC5701143

[76] J. Lian , H. Smidt , R. H. Wijffels , D. Sipkema , Microb. Biotechnol. 2018, 11, 806. 29978601 10.1111/1751-7915.13296 PMC6116740

[77] F. Zhang , Z. Li , C. Chen , H. Luan , R. H. Fang , L. Zhang , J. Wang , Adv. Mater. (Deerfield Beach, Fla.) 2023, 36, 3. 10.1002/adma.202303714 PMC10799182 37471001

[78] X. Liu , H. Xie , S. Roussou , P. Lindblad , Curr. Opin. Biotechnol. 2022, 73, 143. 34411807 10.1016/j.copbio.2021.07.014

[79] S. Gudmundsson , J. Nogales , Mol. BioSyst. 2015, 11, 60. 25382198 10.1039/c4mb00335g

[80] M. Kumar , J. Jeon , J. Choi , S.‐R. Kim , J. Appl. Phycol. 2018, 30, 1735.

[81] I. Kellersztein , D. Tish , J. Pederson , M. Bechthold , C. Daraio , Adv. Mater. 2025, 37, 2413618. 10.1002/adma.202413618 39558799

[82] S. Priyanka , R. Varsha , R. Verma , S. B. Ayenampudi , J. Microbiol. Biotechnol. Food Sci. 2023, 12, e4785.

[83] N. K. AlFadhly , N. Alhelfi , A. B. Altemimi , D. K. Verma , F. Cacciola , Plants 2022, 11, 3063. 36432792 10.3390/plants11223063 PMC9693216

[84] R. A. Ahmed , M. He , R. A. Aftab , S. Zheng , M. Nagi , R. Bakri , C. Wang , Sci. Rep. 2017, 7, 8118. 28808229 10.1038/s41598-017-07540-x PMC5556107

[85] N. Anila , D. P. Simon , A. Chandrashekar , G. Ravishankar , R. Sarada , Photosynth. Res. 2016, 127, 321. 26334599 10.1007/s11120-015-0188-8

[86] J. Cao , J. Wang , S. Wang , X. Xu , J. Med. Food 2016, 19, 111.26653974

[87] S. H. Brawley , N. A. Blouin , E. Ficko‐Blean , G. L. Wheeler , M. Lohr , H. V. Goodson , J. W. Jenkins , C. E. Blaby‐Haas , K. E. Helliwell , C. X. Chan , T. N. Marriage , D. Bhattacharya , A. S. Klein , Y. Badis , J. Brodie , Y. Cao , J. Collén , S. M. Dittami , C. M. M. Gachon , B. R. Green , S. J. Karpowicz , J. W. Kim , U. J. Kudahl , S. Lin , G. Michel , M. Mittag , B. J. S. C. Olson , J. L. Pangilinan , Y. Peng , H. Qiu , et al., Proc. Natl. Acad. Sci. USA 2017, 114, E6361. 28716924 10.1073/pnas.1703088114 PMC5547612

[88] H. Zhang , C. Yang , Mol. Microbiol. 2019, 111, 863. 30656751 10.1111/mmi.14204

[89] Y. Ye , M. Liu , L. Yu , H. Sun , J. Liu , Mar. Drugs 2024, 22, 54. 38393025 10.3390/md22020054 PMC10890015

[90] E. Poliner , E. M. Farré , C. Benning , Plant Cell Rep. 2018, 37, 1383. 29511798 10.1007/s00299-018-2270-0

[91] J. E. Hunter , M. J. Frada , H. F. Fredricks , A. Vardi , B. A. Van Mooy , Front. Mar. Sci. 2015, 2, 81.

[92] B. M. Schieler , M. V. Soni , C. M. Brown , M. J. Coolen , H. Fredricks , B. A. Van Mooy , D. J. Hirsh , K. D. Bidle , ISME J. 2019, 13, 1019. 30607029 10.1038/s41396-018-0325-4 PMC6461841

[93] K. Sun , D. Meesapyodsuk , X. Qiu , Front. Microbiol. 2024, 15, 1381097. 39056009 10.3389/fmicb.2024.1381097 PMC11269151

[94] T. T. Mamo , Y. S. Mekonnen , Appl. Biochem. Biotechnol. 2020, 190, 1147. 31712990 10.1007/s12010-019-03149-0

[95] K. V. Ajayan , M. Selvaraju , P. Unnikannan , P. Sruthi , Int. J. Phytoremediation 2015, 17, 907. 25580934 10.1080/15226514.2014.989313

[96] P. Mougkogiannis , N. R. Kheirabadi , A. Chiolerio , A. Adamatzky , Neuromorph. Comput. Eng. 2024, 4, 014007.

[97] Z. Zeng , Z. Yue , A. Mauroy , J. Gonçalves , Y. Yuan , IEEE Trans. Autom. Control 2024.

[98] Z. Hanusz , J. Tarasińska , Biometr. Lett. 2015, 52, 85.

[99] M. Patrício , F. Ferreira , B. Oliveiros , F. Caramelo , Commun. Stat. Simul. Comput. 2017, 46, 7535.

[100] F. Habibzadeh , J. Korean Med. Sci. 2024, 39, 3. 10.3346/jkms.2024.39.e35 PMC10803211 38258367

[101] A. Ross , V. L. Willson , A. Ross , V. L. Willson , Basic and Advanced Statistical Tests: Writing Results Sections and Creating Tables and Figures, Springer, Berlin 2017, pp. 21–24.

[102] P. Mishra , U. Singh , C. M. Pandey , P. Mishra , G. Pandey , Ann. Card. Anaesth. 2019, 22, 407. 31621677 10.4103/aca.ACA_94_19 PMC6813708

[103] D. C. Sauder , C. E. DeMars , Adv. Methods Pract. Psychol. Sci. 2019, 2, 26.

[104] T. W. MacFarland , J. M. Yates , T. W. MacFarland , J. M. Yates , Introduction to Nonparametric Statistics for the Biological Sciences Using R, Springer, Berlin 2016, 103.

[105] R. Mirabbasi , F. Ahmadi , D. Jhajharia , Hydrol. Res. 2020, 51, 1455.

[106] R. R. Guillard , J. H. Ryther , Can. J. Microbiol. 1962, 8, 229. 13902807 10.1139/m62-029

[107] R. Wördenweber , S. D. Rokitta , E. Heidenreich , K. Corona , F. Kirschhöfer , K. Fahl , J. L. Klocke , T. Kottke , G. Brenner‐Weiß , B. Rost , J. H. Mussgnug , O. Kruse , Limnol. Oceanogr. 2018, 63, 203.

[108] A. Shemi , D. Schatz , H. F. Fredricks , B. A. Van Mooy , Z. Porat , A. Vardi , New Phytol. 2016, 211, 886. 27111716 10.1111/nph.13940

[109] W. Stumm , J. J. Morgan , Aquatic Chemistry: Chemical Equilibria and Rates in Natural Waters, 3rd ed., John Wiley & Sons, Hoboken, NJ, 2013.

[110] T. Proschold , E. H. Harris , A. W. Coleman , Genetics 2005, 170, 1601. 15956662 10.1534/genetics.105.044503 PMC1449772

[111] A. R. Grossman , M. Lohr , C. S. Im , Annu. Rev. Genet. 2004, 38, 119. 15568974 10.1146/annurev.genet.38.072902.092328

[112] G. Lelandais , I. Scheiber , J. Paz‐Yepes , J.‐C. Lozano , H. Botebol , J. Pilátová , V. Žárskỳ , T. Léger , P.‐L. Blaiseau , C. Bowler , F.‐Y. Bouget , J.‐M. Camadro , R. Sutak , E. Lesuisse , BMC genomics 2016, 17, 1. 27142620 10.1186/s12864-016-2666-6 PMC4855317

[113] C. Clerissi , Y. Desdevises , N. Grimsley , J. Virol. 2012, 86, 4611. 22318150 10.1128/JVI.07221-11 PMC3318615

[114] F. Daboussi , S. Leduc , A. Maréchal , G. Dubois , V. Guyot , C. Perez‐Michaut , A. Amato , A. Falciatore , A. Juillerat , M. Beurdeley , D. F. Voytas , L. Cavarec , P. Duchateau , Nat. Commun. 2014, 5, 3831. 24871200 10.1038/ncomms4831

[115] T. Butler , L. Matsakas , M. Amirebrahimi , J. Lundqvist , U. Rova , P. Christakopoulos , Trends Biotechnol. 2020, 38, 606.31980300

[116] M. Puri , N. Arora , I. Haq , K. Permaul , Trends Microbiol. 2023, 31, 872. 36801156 10.1016/j.tim.2023.01.010

[117] S. W. Fox , T. Nakashima , Biosystems 1980, 12, 155. 7397322 10.1016/0303-2647(80)90013-1

[118] E. R. Mattoon , A. Casadevall , R. J. Cordero , Fungal Biol. Rev. 2021, 36, 60.

[119] A. Adamatzky , Interface Focus 2018, 8, 20180029. 30443330 10.1098/rsfs.2018.0029 PMC6227805

[120] A. Adamatzky , Physarum Machines: Computers from Slime Mould , vol. 74, World Scientific, 2010.

[121] A. Tero , S. Takagi , T. Saigusa , K. Ito , D. P. Bebber , M. D. Fricker , K. Yumiki , R. Kobayashi , T. Nakagaki , Science 2010, 327, 439. 20093467 10.1126/science.1177894

[122] T. Nakagaki , H. Yamada , Á Tóth , Nature 2000, 407, 470. 11028990 10.1038/35035159

